# Selective social interactions and speed-induced leadership in schooling fish

**DOI:** 10.1073/pnas.2309733121

**Published:** 2024-04-25

**Authors:** Andreu Puy, Elisabet Gimeno, Jordi Torrents, Palina Bartashevich, M. Carmen Miguel, Romualdo Pastor-Satorras, Pawel Romanczuk

**Affiliations:** ^a^Departament de Física, Universitat Politècnica de Catalunya, Barcelona 08034, Spain; ^b^Departament de Física de la Matèria Condensada, Universitat de Barcelona, Barcelona 08028, Spain; ^c^Institute for Theoretical Biology, Department of Biology, Humboldt-Universität zu Berlin, Berlin 10115, Germany; ^d^Excellence Cluster Science of Intelligence, Technische Universität Berlin, Berlin 10587, Germany; ^e^Institute of Complex Systems (UBICS), Universitat de Barcelona, Barcelona 08028, Spain; ^f^Bernstein Center for Computational Neuroscience, Berlin 10115, Germany

**Keywords:** leadership, social interactions, attention switch, schooling fish, collective behavior

## Abstract

Animals moving together in groups are believed to interact among each other with effective social forces, such as attraction, repulsion, and alignment. Such forces can be inferred using “force maps,” i.e., by analyzing the dependency of the acceleration of a focal individual on relevant variables. Here, we introduce a force map technique suitable for the analysis of the alignment forces experienced by individuals. After validating it using an agent-based model, we apply the force map to experimental data of schooling fish. We observe signatures of an effective alignment force with faster neighbors and an unexpected antialignment with slower neighbors. Instead of an explicit antialignment behavior, we suggest that the observed pattern is the result of a selective attention mechanism, where fish pay less attention to slower neighbors. This mechanism implies the existence of temporal leadership interactions based on relative speeds between neighbors. We present support for this hypothesis both from agent-based modeling as well as from exploring leader–follower relationships in the experimental data.

## Significance

Grouping animals interact socially to move together and make collective decisions. These interactions are typically assumed to be attraction to distant individuals, repulsion from too close neighbors, and alignment among neighbors to coordinate movement directions. Combining analysis of experimental data of schooling fish with agent-based modeling, we find that social interactions are modulated by relative speeds between neighbors, where individuals tend to align only with faster neighbors and ignore slower ones. These selective social interactions can be linked to dynamically changing leader–follower relationships defined by relative speeds of neighbors. Our results provide a deeper understanding of emergent leadership based on variable and adaptive speeds of individuals and a different perspective on mechanisms of movement coordination in natural and artificial systems.

Moving animal groups can exhibit a complex and highly coordinated collective behavior that results in the formation of spatial patterns changing dramatically in time, as observed in fish schools or bird flocks (1, 2). A possible framework for interpreting this complex behavior relies on the concept of effective forces acting on individuals. However, contrary to physical forces, these forces mainly originate from individual decision-making processes and, in particular, do not necessarily fulfill the action-reaction Newtonian principle (3). Depending on their origin, we can classify them into individual forces and social forces. Individual forces are independent of the social environment. They can have a physical explanation related to the mechanics of locomotion, such as propulsion and friction, or be a consequence of nonphysical processes linked to individual movement decision, including intrinsically stochastic behavior and response to environmental cues, such as the presence of tank walls, food sources, or predators. Social forces, on the other hand, emerge from the interactions between the individuals of the group and are usually described in terms of a combination of attraction, repulsion and alignment forces (1, 2, and 4). More specifically, attraction allows individuals to aggregate in groups, repulsion prevents collisions and alignment induces individuals to head in the same direction as their neighbors.

Models of collective motion based on self-propelled particles interacting with neighbors via these three kinds of social interactions are able to reproduce typical features observed in moving animals such as polarized movement, where individuals move globally in the same direction; swarming, where individuals aggregate in a group but move in random directions; and milling, where individuals rotate around a core (5, 6). Although explicit alignment forces are a straight-forward way to induce global orientational state, they are not a necessary condition (3, 7–11).

From an experimental point of view, studies have provided different results regarding the existence of explicit alignment interactions. One possible approach is to postulate a model and optimize its parameters from the experimental data. Lukeman et al. (12) assumed a zonal model, where the different attraction, repulsion, and alignment forces are implemented in concentric layers around the individual, and found that the strength of alignment was comparable to that of attraction. In a similar spirit but without choosing a specific model, Theraulaz et al. (13, 14) proposed symmetrical constraints to identify the contributions of the different interactions and obtained simple analytical functions, assuming that the effect of different variables can be separated as a product of functions. They reported alignment interactions that depend on the relative headings and positions between neighbors. An alternative method is to train a neuronal network with experimental data and infer interactions from it. Heras et al. (15) found evidence of alignment for large neighbor speeds and an antialignment region behind the individual that was associated with possible interactions with the walls.

The most common procedure to measure forces, however, is the use of “force maps,” also known as “averaging method,” where forces are inferred from the dependency of the acceleration with some relevant variables and averaging over all others. While force maps have been used with great success to infer attraction and repulsion forces in moving animal groups (14, 16–20), they are not free from criticism (14, 15, 21): Since force maps can be visualized mainly up to two-dimensions, one either has to visualize cuts of higher dimensional objects that may lack statistics or average over some variables; it is not necessarily straightforward to obtain simple analytical expressions to reproduce the observed force maps; different interactions in a force map can appear mixed, which can make them difficult to identify and disentangle; and different interaction rules may result in similar force maps. The existence of effective alignment forces has been investigated through the analysis of changes in relative heading of neighboring individuals (22). Whereas earlier works failed to identify alignment interactions (16, 17), later studies found some evidence for alignment between neighbors (14, 18, 20).

A common view assumed in most numerical models and experimental analysis is that coherent collective motion emerges spontaneously from symmetrical interactions among identical self-propelled group members. This contrasts with studies of leadership in which the strength of the interactions between pairs is asymmetric and displays a net information flow, sometimes with time delays (23). From the group perspective, one way to represent leader–follower relationships is via complex directed interaction networks (24, 25). Furthermore, leadership structures depend strongly on the characteristics of the species but also on many individual factors such as information, physiological state, dominance, and navigational competence. In previous works on birds (24), bats (26), and fish (16, 17, 25, 27–30), information flow was identified to naturally occur from influential individuals at the front of the group toward following individuals located further back. Leadership was also observed to depend on average individual speeds of previous solo flights for homing pigeons (31) and for schooling fish (29, 32, 33). Furthermore, deep learning tools have provided evidence that individuals are more influenced by neighbors moving at high speeds (15, 30).

Here, we review the concept of force maps and introduce a more natural alignment force map in Cartesian coordinates that depends on relative velocities of the focal individual with respect to its neighbors. Using a simple model of collective motion with social and individual forces, we demonstrate the utility of the force maps to capture the attraction, repulsion, and alignment forces. Equipped with the proposed alignment force map, we apply it to experimental data of schooling fish and obtain two distinct behaviors: alignment with a neighbor that moves faster than the individual and an unexpected region of apparent antialignment when a neighbor moves at slower speeds than the individual. In order to explore the origin of this antialignment signal, we investigate an agent-based model with explicit alignment and antialignment forces and show that it fails to reproduce realistic behavioral dynamics. Instead, we are able to reproduce the qualitative behavior of the experimental data with a selective interactions model, where individuals only interact with neighbors at faster speeds and ignore neighbors with slower speeds. We further examine the selective-interaction hypothesis by studying the effect of leadership at the level of force maps and demonstrate that a follower displays a pure alignment force and a leader effectively a pure antialignment force. Finally, we validate the hypothesis that individuals with slower and faster neighbors are leaders and followers respectively, studying time-delayed correlations between the velocities of a focal fish and its neighbor.

## Results

### Force Maps.

The force map method allows inferring forces from a system. It assumes the system has several force terms depending on sets of independent variables, and that the average in time of each force term is zero. Consider we want to infer a force term $\vec{f}\left(x\right)$ as a function of only the variable x. We can achieve this from Newton’s second law by studying the acceleration depending on x while averaging over all other variables in the system, $\vec{a}\left(x\right)$ (16). Similar procedures have also been employed in deriving effective equations of motion for motile cells (34). Working in units of mass m=1, Newton’s second law will become $\vec{f}\left(x\right)≃\vec{a}\left(x\right)$, as all other forces will cancel out in the average. In practice, this approach is implemented by binning over the whole dataset the relevant variable x as ${\left\{x_{k}\right\}}_{k=1,\ldots ,M}$, where k is the index of the bin, and averaging the values of the acceleration on that bin, $\vec{f}\left(x_{k}\right)≃{\langle \vec{a}\left(x\right)\rangle }_{x\in x_{k}}$.

#### Attraction–repulsion force map.

**Fig. 1.**
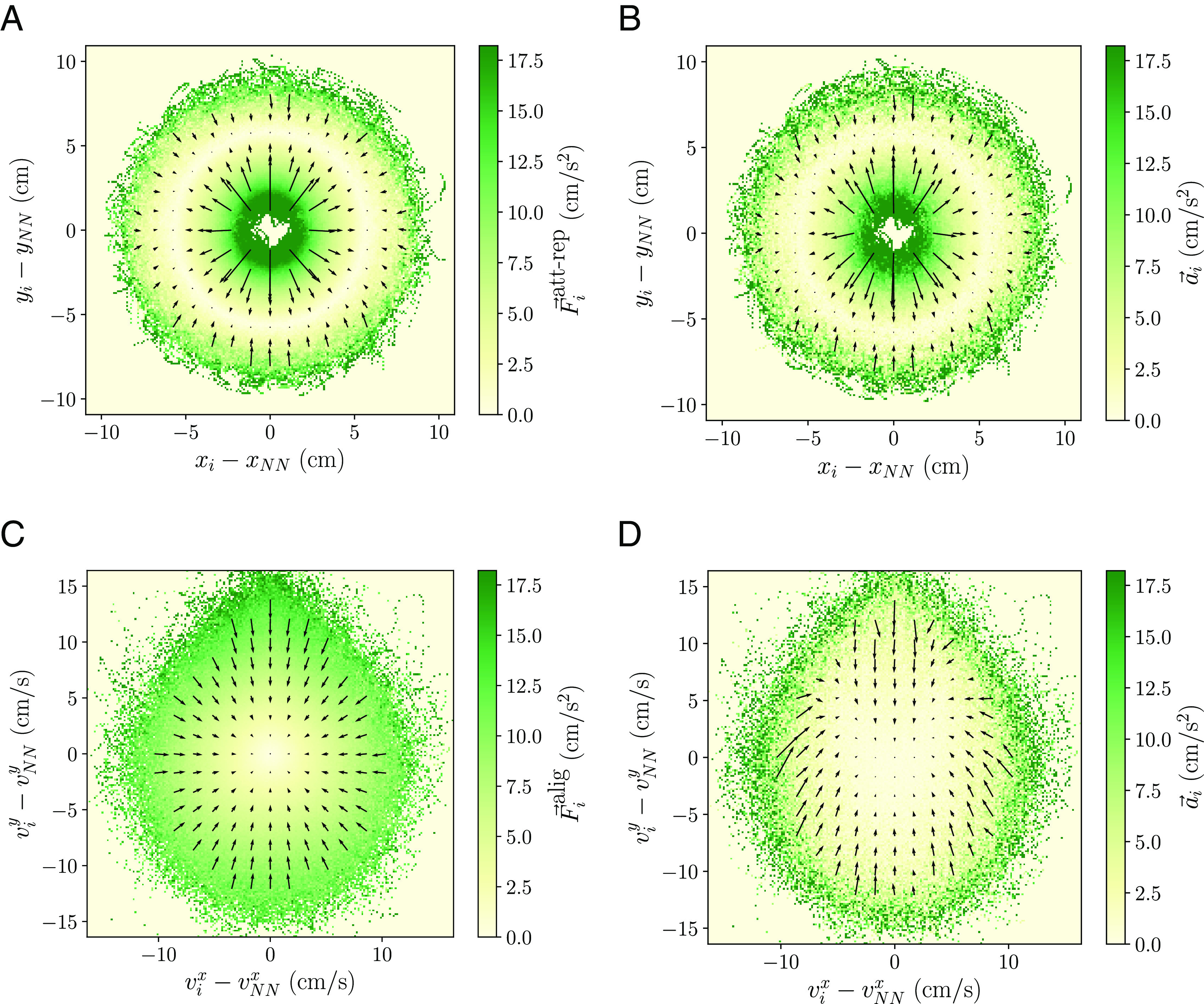
Attraction–repulsion and alignment force maps for the standard model. The force maps are able to recover qualitatively the behavior of the corresponding forces. Average (*A*) attraction–repulsion force and (*B*) acceleration (attraction–repulsion force map) acting on an individual i depending on its relative position with the nearest neighbor NN, ${\vec{x}}_{i}-{\vec{x}}_{\mathrm{NN}}$. We observe for small distances repulsion, for intermediate distances no net force and for large distances attraction. Average (*C*) alignment force and (*D*) acceleration (alignment force map) acting on an individual i depending on its relative velocity with the nearest neighbor NN, ${\vec{v}}_{i}-{\vec{v}}_{\mathrm{NN}}$. In all plots, the y-axis is along the direction of motion of the focal individual, the colormap displays the modulus of the force and the arrows its components. Forces are expressed in units of mass m=1. We employ values for all focal individuals and time steps in the simulation.

This force map was introduced both by Katz et al. (16) and Herbert-Read et al. (17). It obtains the attraction and repulsion forces from the average acceleration ${\vec{a}}_{i}$ of an individual i as a function of its position relative to the nearest neighbor NN, ${\vec{x}}_{i}-{\vec{x}}_{\mathrm{NN}}$, assuming this neighbor dominates the interaction. We can demonstrate it with a simple model of schooling fish, which we refer to as standard model. In this model, individuals interact with some social forces, described by attraction–repulsion and alignment terms, and respond to some individual forces, represented by a friction–propulsion term to drive the motion and noise (for more details, see *Materials and Methods*). Employing simulations of the model, we display in Fig. 1*A* the average attraction–repulsion force acting on a focal individual depending on its position relative to the nearest neighbor. We use values for all focal individuals and time steps (counts are shown in *SI Appendix*, Fig. S1*A*). The components of the force are displayed with arrows and the modulus with the colormap. The y-coordinate is oriented along the direction of motion of the focal individual. We observe for near distances arrows point outward, indicating the focal fish experiences a repulsive force away from the neighbor. For intermediate distances, there is an area with no net force (the equilibrium distance). Finally, for far away distances arrows point inward, signaling an attractive force toward the neighbor. Now in Fig. 1*B*, we display the attraction–repulsion force map given by the average acceleration for an individual depending on its relative position with the nearest neighbor. Indeed, we observe it recovers satisfactorily the attraction–repulsion force. This procedure works adequately because other forces do not depend directly on the relative position of the individual with the nearest neighbor and tend to cancel out in average (*SI Appendix*, Fig. S1 *B* and *C*).

We note that previous works on the attraction–repulsion force map employed the reversed coordinates ${\vec{x}}_{\mathrm{NN}}-{\vec{x}}_{i}$, but they typically separated the acceleration components in different plots and did not display the force with arrows (except in ref. 20). Because here instead we display the force as a vector, we find it more natural to represent it analogously to a force field from physics, where the force at a point indicates the force a particle would feel at that point. Furthermore, we also want to remark that, in absence of other forces, a trajectory observed in the force map is more complex than just following the direction of the arrows, as there will be rotations caused by the y-axis pointing toward the direction of motion of the focal individual.

#### Alignment force map.

Previous works used force maps for the alignment force that depend on angular differences as well as on relative positions, which make the alignment force more difficult to disentangle from the attraction and repulsion forces. Here, instead, we introduce a more natural force map for alignment in Cartesian coordinates obtained from calculating the average acceleration ${\vec{a}}_{i}$ of an individual i as a function of its relative velocity with the nearest neighbor NN, ${\vec{v}}_{i}-{\vec{v}}_{\mathrm{NN}}$. This approach is directly inspired from the expression of the alignment force between neighbors in our standard model (see Eq. **2** in *Materials and Methods*). It naturally accounts for the alignment of the full velocity vectors of neighboring individuals, thus not only their direction of movement but also of their speeds.

In Fig. 1*C*, we show the average alignment force acting on a focal individual depending on its relative velocity with the nearest neighbor for the standard model. We employ values for all focal individuals and time steps (counts are shown in *SI Appendix*, Fig. S2*A*). The components of the force are displayed with arrows and the modulus with the colormap. The y-coordinate is oriented along the direction of motion of the focal individual. We observe the force vectors point inward and increase with the velocity differences. This results in the force term decreasing the relative velocity and aligning the velocity of the focal individual with that of its neighbor. In Fig. 1*D*, we show the alignment force map given by the average acceleration in this representation. This force map also recovers successfully the qualitative behavior of the alignment force, with arrows pointing inward (this is clearly observed looking at the projection of the acceleration in the radial direction of the force map in *SI Appendix*, Fig. S3*A*). Again, this works because other forces of the model do not depend directly on the relative velocity of the individual with the nearest neighbor and have small contributions in this representation (*SI Appendix*, Fig. S2 *B* and *C*).

### Experimental Force Maps.

Equipped with the force maps technique outlined above, we are now able to explore experimental data of schooling fish. With this purpose, we analyze the motion of N=39 individuals of the species black neon tetra Hyphessobrycon herbertaxelrodi, a highly social fish that tends to form polarized, compact, and planar schools, freely swimming in an approximately two-dimensional experimental tank (see *Materials and Methods* for experimental details).

**Fig. 2.**
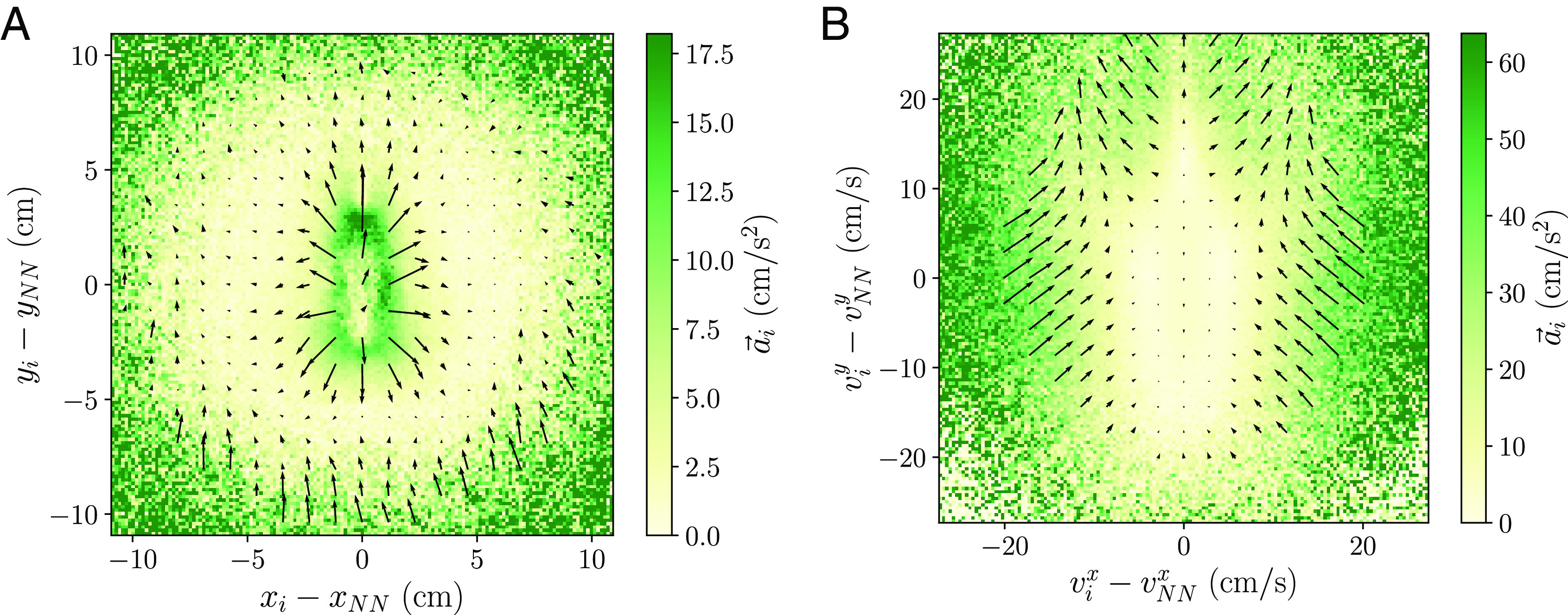
Force maps for the experimental data: (*A*) attraction–repulsion and (*B*) alignment force map. While the attraction–repulsion force map is similar to the standard model, the alignment force map displays a striking different behavior: In the lower-half region, arrows point inward indicating alignment, but in the upper-half region, arrows point outward indicating antialignment.

We start with the attraction–repulsion force map in Fig. 2*A* (the counts of the force map are shown in *SI Appendix*, Fig. S4*A*). We see this force map has a similar qualitative behavior to the attraction–repulsion force for the standard model (Fig. 1*A*), with repulsion for short distances, no net force for intermediate distances and attraction at large distances.

Now we turn our attention to the alignment force map obtained from experimental data, displayed in Fig. 2*B* (the counts are shown in *SI Appendix*, Fig. S4*B*). Contrary to the alignment force from the standard model (Fig. 1*C*), we can identify two qualitatively different behaviors depending on the sign of the difference of velocities between an individual and its nearest neighbor in the direction of motion of the individual (y-axis). If the neighbor is moving at faster speeds than the individual (lower-half part), we observe alignment interactions with the acceleration pointing inward. However, when the neighbor moves at slower speeds than the individual (upper-half part), the acceleration points outward and indicates a surprising antialignment interaction, with fish turning away from neighbors. This observation is made more apparent with the projection of the acceleration in the radial direction of the force map, in *SI Appendix*, Fig. S3*B*.

We find that the qualitative behavior of the alignment and antialignment regions in the alignment force map is robust against a variety of factors: different number of fish (*SI Appendix*, Fig. S5); different distances between neighbors (*SI Appendix*, Fig. S6), which tests for possible effects originating from the attraction or repulsion regimes in the attraction–repulsion force; different distances to the tank walls (*SI Appendix*, Fig. S7), which discards possible interactions with the walls; for the case the fish is approaching or moving away from the neighbor (*SI Appendix*, Fig. S8); for different heading orientations between the fish and its neighbor (*SI Appendix*, Fig. S9); and for different velocities of the focal individual (*SI Appendix*, Fig. S10). Only when differentiating the case where the neighbor moves at faster or slower speeds (*SI Appendix*, Fig. S11), we observe exclusively alignment or antialignment interactions in the expected regions of the map.

### Explicit Antialignment Model.

We find the standard model of schooling fish reproduces properly the attraction–repulsion force map in the experimental data of schooling fish, but is unable to account for the alignment force map. To correct this, we now consider a model variation implementing explicitly the alignment force observed experimentally. We do so by defining an alignment force given by an interpolation of the values in the experimental alignment force map, which is previously smoothed with a Gaussian filter of window σ=2. All other forces and parameters are kept the same as in the previous section.

**Fig. 3.**
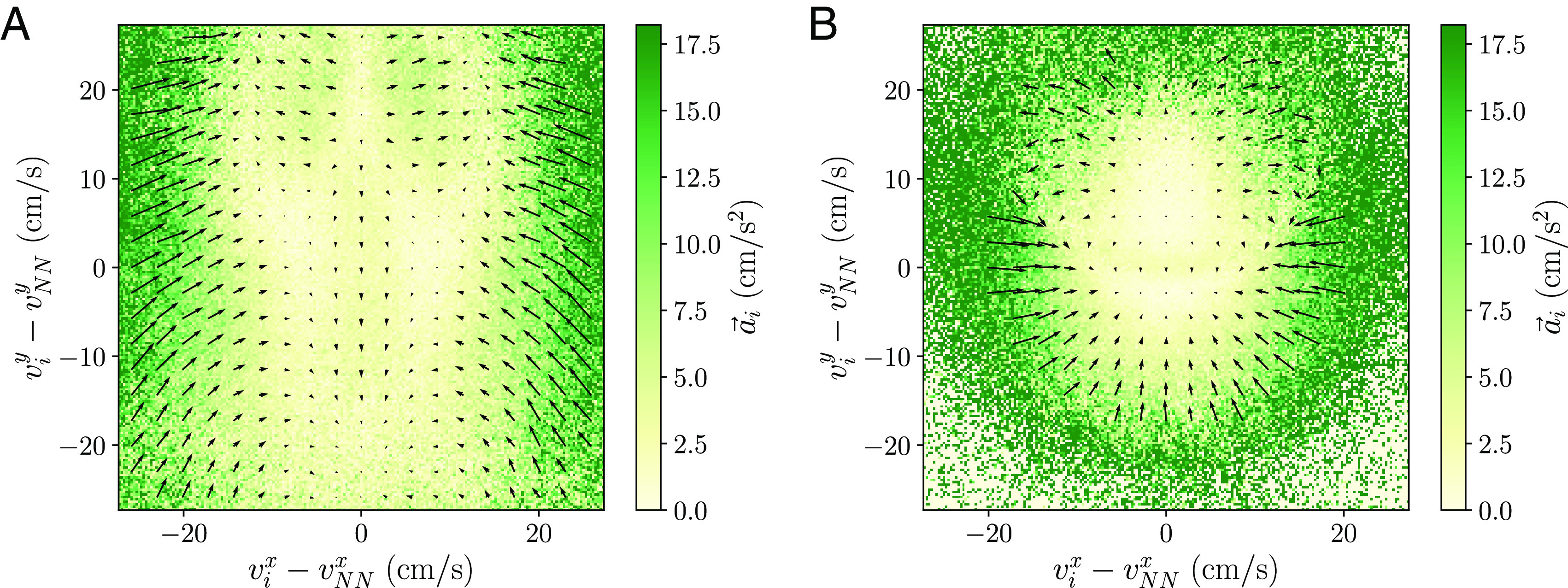
Model variations with alignment and antialignment regions in the alignment force map. Alignment force maps for (*A*) the explicit antialignment model and (*B*) the selective interactions model.

Despite the model reproduces qualitatively the expected alignment force map (Fig. 3*A* and *SI Appendix*, Figs. S3*C* and S12), the dynamics is unrealistic. When an individual is moving faster than its neighbors, it is located in the antialignment region of the force map and the explicit antialignment force causes the individual to accelerate in the forward direction, which in the absence of other forces will result in an even larger speed. This behavior is observed in the simulations of the model (Movie S3), where some individuals acquire large speeds and move toward the front of the group. However, this is not found in the experimental data, and there is no biological motivation for fast fish to waste energy by increasing their speed further.

### Selective Interactions Model.

The nonphysical and nonbiological features of the explicit antialignment model suggest that the antialignment region observed in the alignment force map of the experimental data may not originate from an explicit antialignment rule, but it could instead emerge from some other interaction that appears effectively as an antialignment force.

To explore this idea, we first study the effect of a persistent random force in the alignment force map. We propose a variation of the standard model, in which individuals have some probability rate r to switch off their social interactions and feel instead a persistent force in a fixed random direction with a given amplitude A for some time τ. Biologically, this can be motivated as an individual fish may decide to follow private information based on environmental cues and temporarily ignore social cues. Depending on the relative strength of the persistent random force compared to the alignment force, we observe two different outcomes. When the alignment force dominates the persistent random force, we obtain an alignment force map that is still full alignment (*SI Appendix*, Fig. S13*B*). However, the persistent random force contributes to the total force with antialignment (*SI Appendix*, Fig. S13*D*). We can understand this as in a group with overall high directional alignment, a persistent “private” force acting temporarily in a fixed random direction will tend to make the focal fish turn away from their neighbor’s direction and appear effectively as an antialignment interaction. Naively, one could expect that in the regime when the persistent random force dominates the alignment force, the resulting alignment force map would be antialignment. However, that is not the case as there is not anymore ordered collective motion and the force map is essentially noise.

To connect this idea with the observed experimental alignment force map, we propose another variation of the standard model, a selective interactions model where individuals pay attention primarily to neighbors exhibiting strong movement cues. We assume that individuals interact socially with neighbors that move at a speed larger than their own, and switch off social interactions with neighbors that move at a smaller speed (Movie S4). The weights in the social interactions are calculated as in the standard model (*Materials and Methods*) but considering only faster neighbors. We display the alignment force map in Fig. 3*B* (*SI Appendix*, Figs. S3*D* and S14). We see this model reproduces the features observed in the experimental data, with alignment for faster neighbors and antialignment for slower neighbors. The emergence of antialignment for slower nearest neighbors in this model can be traced back to the attraction–repulsion force (*SI Appendix*, Fig. S14). Although this force is switched off for slower neighbors, the focal individual will continue to interact with other nonnearest faster neighbors. Thus, when we construct the alignment force map for the slower nearest neighbors, the actual dynamics of the focal agent is driven by attraction to farther away neighbors, which may be located at random relative positions. This force then can act in any direction and, therefore, appears effectively as a persistent random force. As we found previously, this results in individuals on average turning away from their nearest neighbor and displaying apparently an antialignment interaction.

### Comparison of Model Observables.

**Fig. 4.**
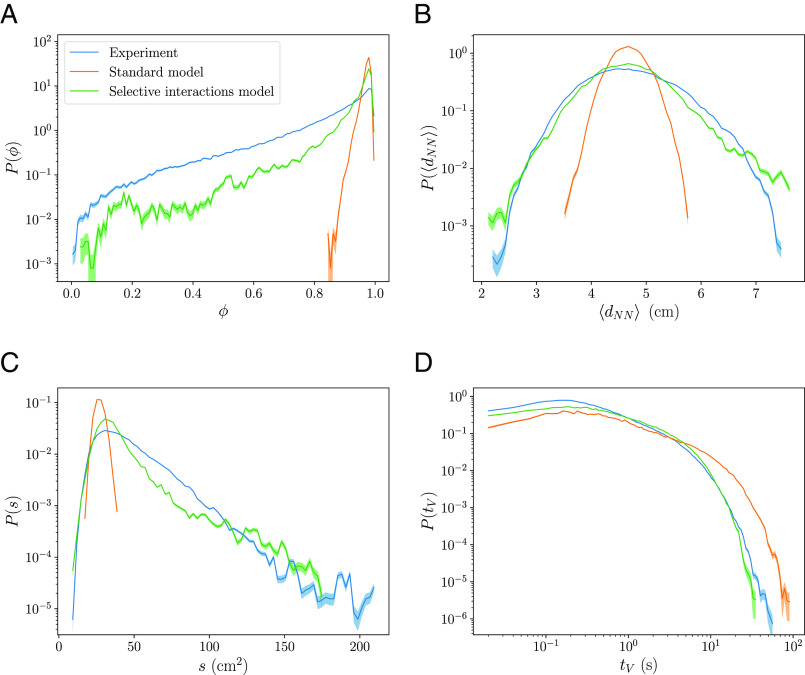
The selective interactions model is more similar to the experimental data than the standard model for a set of observables. Probability density functions (PDF) of (*A*) polarization ϕ, (*B*) instantaneous average nearest neighbor distance $\langle d_{\mathrm{NN}}\rangle$, (*C*) area of the convex hull normalized by the number of individuals s and (*D*) contact duration time between Voronoi neighbors $t_{V}$. The error bands are calculated from the SD of a Bernoulli distribution, with the probability given by the fraction of counts in each bin.

Our analysis based on the alignment force map suggests the selective interactions model as a more accurate depiction of the experimental data than the standard model. Here, we extend our investigation by comparing the models and the experimental data with a set of observables (Fig. 4 and *SI Appendix*, Fig. S15). The polarization ϕ, defined as

$$\begin{array}{c} \phi \equiv |\frac{1}{N}\sum_{i}\frac{\vec{v}}{v}|, \end{array}$$

is a measure of the group order that tends to 1 if all individuals move in the same direction, and decreases when they move in increasingly different directions. The nearest neighbor distance averaged for all individuals $\langle d_{\mathrm{NN}}\rangle$ is a measure of the cohesiveness of the group. The convex hull of the group is defined as the smallest convex polygon that contains all individuals. Its area normalized by the number of individuals s is a measure of the extension occupied by the group. The contact duration time between Voronoi neighbors $t_{V}$ is a measure of the persistence of the local neighborhood structure. In addition, we also consider individual speeds $v_{i}$ and turning rates $\omega_{i}$.

The selective interactions model outperforms the standard model in its ability to reproduce the various experimental observables: It is on average less ordered by displaying a broad distribution of order parameter values (with more low polarization values in Fig. 4*A*); it exhibits a larger variability in the nearest neighbor distances and in individual speeds (wider PDFs in Fig. 4*B* and *SI Appendix*, Fig. S15*B*, respectively), more extended groups (with higher values for the convex hull areas in Fig. 4*C*) and larger rates of neighbors shuffling (with shorter contact duration times of Voronoi neighbors in Fig. 4*D*). On the other hand, individual turning rates are similar for the models and the experimental data (*SI Appendix*, Fig. S15*A*), despite the unbounded space of the models results in less large turning rates. We also observe in *SI Appendix*, Fig. S15*C* that both models display oscillations in individual speeds that resemble the oscillations in the experimental data, which are a consequence of the burst-and-coast mechanism of fish locomotion (35–37).

The similarities in a set of observables between the selective interactions model and the experimental data provide additional support for the selective interactions hypothesis. However, given the model’s parsimonious approach, we do not anticipate it to account for many specific details of the system.

### Leadership and Alignment.

The above results suggest that the features of the experimental alignment map may be explained by selective social interactions with faster individuals. In this section, we want to validate this hypothesis, connecting it to the possible presence of leader–follower relationships.

**Fig. 5.**
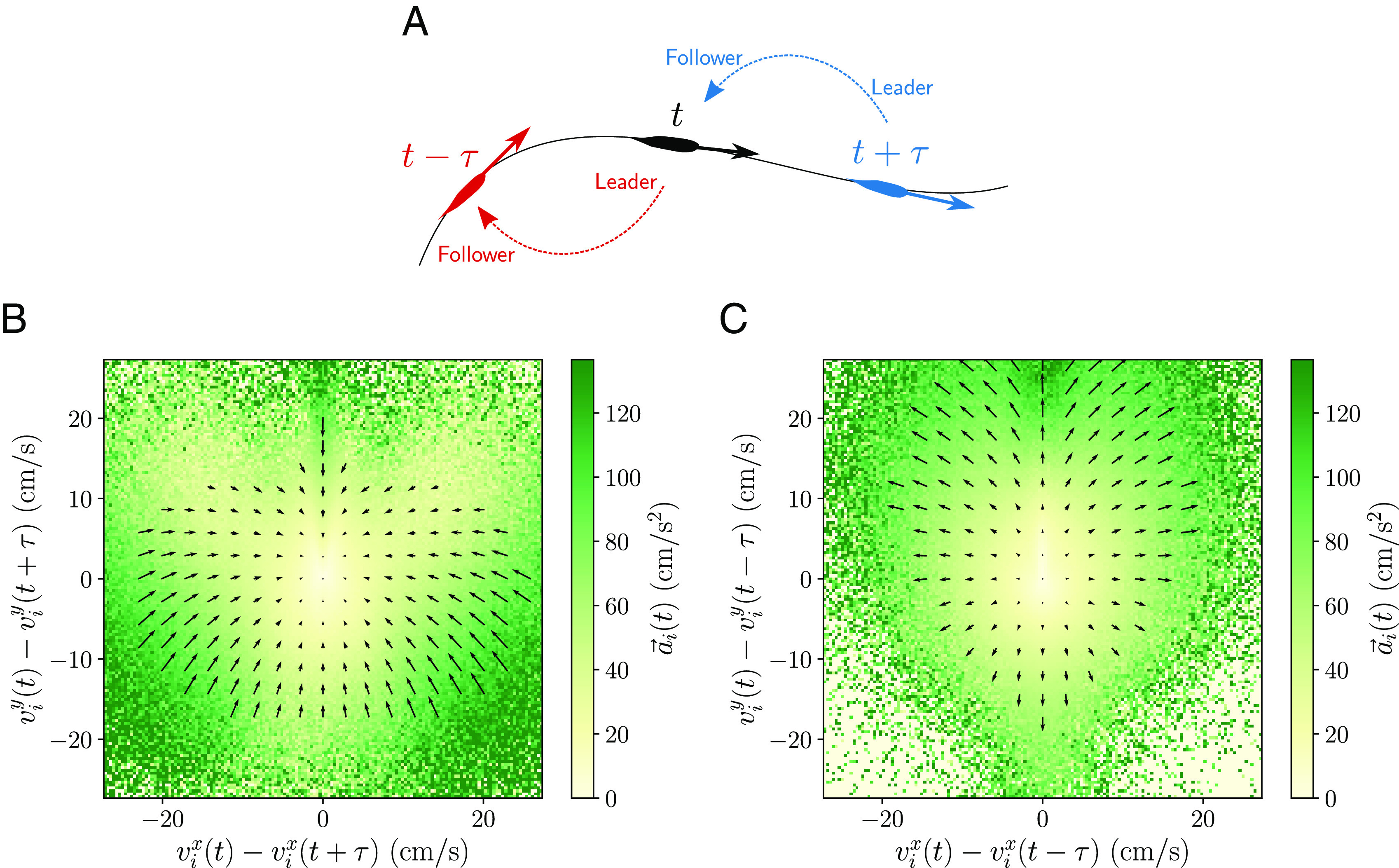
Surrogate data of the experimental school with known leader–follower interactions. (*A*) We compare the individual i with itself at a delayed time τ. (*B*) and (*C*) Alignment force maps comparing the individual i with itself at a (*B*) positive and (*C*) negative delayed time τ=0.2 s. When the delay is positive, the individual at present acts as a follower and we see pure alignment. When the delay is negative, the individual at present acts as a leader and we see pure antialignment.

We start by constructing surrogate data where we know a priori the flow of information between leaders and followers and use the alignment force map to explore it. To achieve this, we use the following trick in the experimental data: For the focal individual, we take the individual i at the present time t, while for the neighbor, we consider the same individual i at a delayed time t+τ. The situation is depicted in Fig. 5*A*. In the case of a positive delay τ, the direction of the individual at present will follow that of the individual in the future after the delayed time, such that the individual at present can be interpreted as the follower within this pair. Now, when we observe the alignment force map for the individual at present (Fig. 5*B* and *SI Appendix*, Figs. S3*E* and S16), we find alignment in all regions. This suggests a follower aligns with a leader. In the case of a negative delay, we have the opposite situation and the direction of the individual at present is adopted by the individual in the past after the delayed time and thus the individual at present can be interpreted as the leader. In the alignment force map (Fig. 5*C* and *SI Appendix*, Figs. S3*F* and S16), this results in antialignment in all regions, i.e., a leader appears to antialign with a follower. However, this does not necessarily imply explicit antialignment, but originates rather from the leader ignoring the follower and moving in a different direction.

**Fig. 6.**
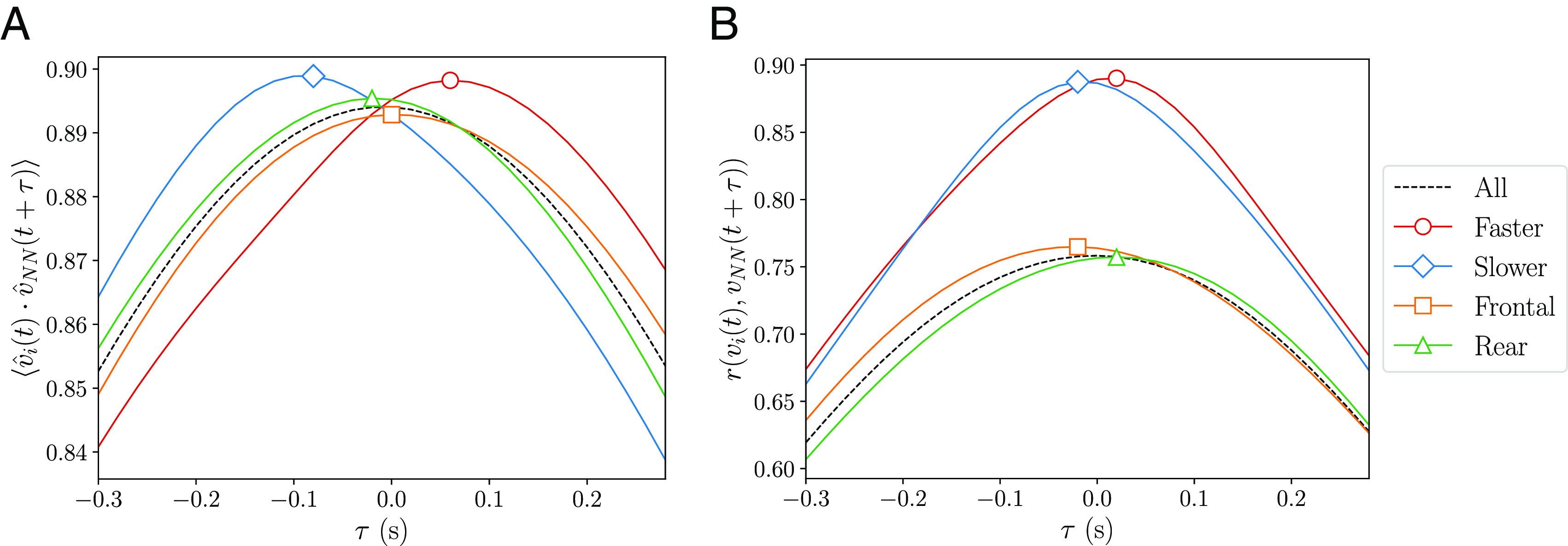
Relative speeds in neighbors describe better leadership in schooling fish than frontal distances along the direction of motion of the individual. Correlations in (*A*) the orientation and (*B*) speed (Pearson correlation coefficient r) between an individual i and its nearest neighbor NN at a delayed time τ for the experimental data. We filter for different states of the individual compared to the neighbor (legend). The markers denote the maximum of each line. We find correlations are higher for faster and slower individuals than frontal and rear individuals. Frontal and rear individuals are more similar to the baseline correlations given by all individuals.

Extrapolating this analysis to the experimental alignment force map (Fig. 2*B*) hints to the idea that individuals act as leaders when moving faster than their neighbors (antialignment region), while they act as followers when moving slower than their neighbors (alignment region). We verify this idea by calculating correlations between the velocities of the individual and the nearest neighbor at a delayed time in the experimental data. We consider two types of correlations, in the orientation given by $\langle {\hat{v}}_{i}\left(t\right)\cdot {\hat{v}}_{\mathrm{NN}}\left(t+\tau \right)\rangle$ (24) (Fig. 6*A*) and in the speed given by the Pearson correlation coefficient $r(v_{i}\left(t\right),v_{\mathrm{NN}}\left(t+\tau \right))$ (16) (Fig. 6*B*). Both measures are computed employing all time frames t. In the presence of leadership interactions, a follower requires some time to react and copy the leader. This results in these correlations having a higher value at that delayed time, provided the frame rate is higher than the reaction time of the follower. If the maximum occurs for a positive delay τ, this implies the individual is the leader. While for a negative delay, the individual is the follower. Moreover, the value of the maximum reflects the amount of information transmitted by the leadership flow.

We are interested in studying correlations differentiating by the state of the individual. In Fig. 6, the red and blue lines display the correlations performed for individuals faster and slower than their neighbor, respectively. Indeed, we see in both correlations that faster individuals have a maximum for positive delays, which means they are leaders. The opposite occurs with slower individuals, which act as followers. We also compare this measure of leadership with the commonly used measure of frontal distances along the direction of motion of the individual (24, 38), filtering for frontal and rear individuals (orange and green). In fact, we find correlations have higher values for faster and slower individuals than for frontal and rear individuals, while frontal and rear individuals are more similar to the base line correlations considering all individuals (black dashed line). This indicates leadership in neighbors is better described by relative speeds rather than with frontal distances along the direction of motion. Note also that the faster and slower curves are not necessarily symmetrical along the y-axis, as several individuals can have the same nearest neighbor. The same occurs for frontal and rear, but in addition, a frontal and rear individual in the direction of motion of the individual can also be frontal and rear respectively in the direction of motion of the neighbor.

As an example of this phenomenon, in Movie S5 we display a sample of a recording where we observe how the focal individual (green) tends to align with faster neighbors (blue) and ignore slower neighbors (red).

## Discussion

In this paper, we have introduced a force map to study the alignment force. Using this force map together with the attraction–repulsion force map, we have been able to capture and distinguish the attraction–repulsion and alignment forces in a simple model of schooling fish. Applying it to experimental data, we found a robust signature of alignment of the focal individual with faster neighbors and antialignment with slower neighbors. Instead of explicit antialigning behavior, we suggest that the signatures of antialignment are the result of a selective attention mechanism, where fish pay less attention and, thus, have weaker interactions with slower neighbors. We were able to find support for this hypothesis by analyzing the effective interactions in an agent-based model with selective social forces. While inferences from interactions in force maps are unlikely to be unique (14), we tested the hypothesis building a connection between the alignment patterns and leadership interactions. We show that followers display only alignment force and leaders only antialignment force. We then verified in the experimental schooling fish that faster and slower individuals are leaders and followers respectively with their nearest neighbors by calculating correlations between the velocities of the individual and the nearest neighbor at a delayed time. This measure of leadership is found to be more statistically significant than the traditionally used in the literature of frontal distances along the direction of motion (24, 27, 38).

Our findings of selective social interactions based on relative individual speeds provide further evidence of attention switch mechanisms previously described in collective behavior (3, 39–42), as well as in the similar field of animal contests over resources (43, 44). Attention switch mechanisms are believed to reduce the cognitive load of individuals and facilitate efficient and accurate decision-making processes (39–41). Our experimental species black neon tetra displays a strong schooling behavior. Thus we expect our results to be representative of other fish species forming highly polarized schools. In any case, the methods introduced in this work are general and therefore, applicable to other scenarios.

If we interpret social interactions between individuals as a method to transfer information across the group, these results suggest higher relevance of information provided by faster-moving individuals (30). This makes biological sense as informed individuals acting on privately acquired information, such as perception of a predator or food, are expected to move at higher speeds (25). More generally, motion cues are believed to play a major role in visual sensory processes of organisms, conveying information that can directly impact the individual, such as in navigation, foraging, courtship or predator avoidance (40, 45); but also as faster movement cues, embedded in a background of slower moving ones, are perceptually more salient (39, 46, 47). Apart from vision, the lateral line, with which fish detect movement, vibration, and pressure gradients in the surrounding water, is also reactive to neighboring individual speeds (48, 49). It is important to emphasize that, in the context of these latter hypotheses, the reported selective interactions would not be attributed to an adaptive selective attention mechanism, but would instead emerge from cognitive constraints related to receiving information from slower-moving individuals.

While previous research suggests that individual speeds play an important role in shaping collective movement, and that leader–follower interactions are mediated by speeds, the proposed mechanism was based on individuals with different average speeds, assuming homogeneous and constant social interactions (29, 31–33). Having different average speeds induces sorting of individuals and ultimately give rise to the well-established leadership of frontal distances along the direction of motion (18). With the current work, we take a step forward connecting speed and leadership. The presence of selective social interactions based on instantaneous relative speeds implies a dynamic modulation of social interactions, which not only amplifies the importance of individual speed, but also provides a mechanism for dynamic adaptation of leader–follower relationships on short time-scales independently of the average preferred speed of individuals.

In the calculation of force maps, we considered only the relative position and velocity of a single, nearest neighbor. While this is supported by 1 to 2 influential neighbors identified in previous work on fish schools (17, 50, 51), one could, in principle, also use the relative position and velocity of all Voronoi neighbors (*SI Appendix*, Fig. S17). While we observe partial screening of attractive interactions in the attraction–repulsion force map, the force maps with Voronoi neighbors resemble qualitatively the force maps with the nearest neighbor. This suggests that the nearest neighbor dominates the interactions. We also find support for this idea when we explored simulations with individuals interacting only with the nearest neighbor and found similar results (*SI Appendix*, Fig. S18) and for simulations with individuals interacting with Voronoi neighbors without weights that resulted in more inadequate force maps with some screening effects.

Force map techniques to infer underlying interactions are straightforward and easy to apply. However, interpreting the results requires caution, and implementing certain validation procedures is advised. In this work, we have presented a force map to extract the alignment force between individuals in experimental data of schooling fish. A first step in validating the results involves ensuring the force map is unaffected by other interactions. We approached this by identifying potential confounding factors such as the influence of tank walls, the attraction–repulsion force, and the group’s density. We then demonstrated that the results were consistent under changing conditions of distances to the walls, distances, and orientations to neighbors and employing schools with differing numbers of fish. A secondary validation measure consists of applying the force map to simulations of models with controlled interactions and comparing the findings. We found that a simple model of schooling fish does not reproduce the experimental signatures and some modifications had to be introduced. Finally, to conclusively validate the results, it is recommended to employ other independent methods to infer interactions. In our study, we accomplished this by analyzing leader–follower relationships. Other possible methods include fitting simple expressions from symmetrical constraints and simplifying hypotheses (13), and training neural networks (15).

Our results showcase how selective social interactions may lead to unexpected signatures at the level of averaged behavioral responses to social cues. It is of relevance for quantitative analysis of social interactions across taxa, as it demonstrates that extreme caution is needed when extracting behavioral algorithms from aggregated data such as force maps, in particular in the context of complex self-organized collective behaviors. Here, a combination of quantitative data as well as theoretical modeling taking into account properties and constraints of perception will provide deeper insights into social interactions and functional aspects of collective behavior with respect to transfer and distributed processing of information. More specifically, selective social interactions depending on relative speeds likely play an important role for different grouping phenomena (3) and provide an explicit mechanism on how speed may be a core variable at the individual level affecting the dynamics and the structure of animal collectives (32, 33, 52–54).

## Materials and Methods

### Standard Model.

We consider a model of schooling fish in two-dimensions with N individuals where each individual i is subject to a total force ${\vec{F}}_{i}$ given by social forces, represented by attraction–repulsion ${\vec{F}}_{i}^{\text{att-rep}}$ and alignment ${\vec{F}}_{i}^{\text{alig}}$ forces, and individual forces, consisting of a friction–propulsion force ${\vec{F}}_{i}^{\text{fric-prop}}$ and noise ${\vec{F}}_{i}^{\text{noise}}$ that capture other unknown forces (55):

$$\begin{array}{c} {\vec{F}}_{i}={\vec{F}}_{i}^{\text{att-rep}}+{\vec{F}}_{i}^{\text{alig}}+{\vec{F}}_{i}^{\text{fric-prop}}+{\vec{F}}_{i}^{\text{noise}}. \end{array}$$

For simplicity, we use a first-order approximation for the different force terms.

The attraction–repulsion force acting on an individual i due to the neighbor j reads:

[1] $$\begin{array}{c} {\vec{F}}_{ij}^{\text{att-rep}}=-k\left(d_{ij}-d_{0}\right)\frac{{\vec{x}}_{i}-{\vec{x}}_{j}}{d_{ij}}, \end{array}$$

where $d_{0}$ is the equilibrium distance between neighbors, $d_{\mathrm{ij}}=|{\vec{x}}_{j}-{\vec{x}}_{i}|$ is the distance between individuals i and j, ${\vec{x}}_{i}$ is the position of individual i and *k* = *k*_rep_ for *d*_ij_ ≤ *d*_0_ and *k* = *k*_att_ for *d*_ij_ > *d*_0_. This force acts as a restoring force toward the equilibrium distance between the neighbors.

The alignment force experienced by an individual i due to its neighbor j is given by ref. 56:

[2] $$\begin{array}{c} {\vec{F}}_{ij}^{\text{alig}}=-\mu ({\vec{v}}_{i}-{\vec{v}}_{j}), \end{array}$$

where μ is the alignment constant and ${\vec{v}}_{k}$ are individual velocities. This force acts as a restoring force for the focal individual toward reaching the same speed and heading as its neighbor.

We use interactions with Voronoi neighbors, which are a good approximation to visual interaction networks and can be calculated efficiently (56–59), and we average the social forces, ${\vec{F}}_{ij}^{\text{social}}={\vec{F}}_{ij}^{\text{att-rep}}+{\vec{F}}_{ij}^{\text{alig}}$, for the different neighbors with weights given by the inverse of their metric distance $d_{\mathrm{ij}}$:

$$\begin{array}{c} {\vec{F}}_{i}^{\text{social}}=\sum_{j}w_{ij}{\vec{F}}_{ij}^{\text{social}}, \end{array}$$

where $w_{\mathrm{ij}}=\frac{1}{d_{\mathrm{ij}}}$. With this choice of weights, closer neighbors exert more influence, a result observed in previous studies (15, 17, 25, 60).

For the individual forces, we use a friction–propulsion term (56):

$$\begin{array}{c} {\vec{F}}_{i}^{\text{fric-prop}}=-\frac{v_{i}-v_{0}}{\tau }{\hat{v}}_{i}, \end{array}$$

where $v_{0}$ is the fish preferred speed and τ is the speed relaxation time. This introduces a self-propulsion force for the fish to move with a speed around $v_{0}$, with τ being the typical timescale of velocity adaptation. The noise force corresponds to active fluctuations (56, 61), with independent noise terms acting on the direction of motion ${\hat{v}}_{i}$ and its perpendicular direction ${\hat{\varphi }}_{i}$:

$$\begin{array}{c} {\vec{F}}_{i}^{\text{noise}}=\sigma_{v}\xi_{v}(t){\hat{v}}_{i}+\sigma_{\varphi }\xi_{\varphi }(t){\hat{\varphi }}_{i}, \end{array}$$

where $\sigma_{v}$ and $\sigma_{\varphi }$ are the noise constants, and $\xi_{v}\left(t\right)$ and $\xi_{\varphi }\left(t\right)$ are independent Gaussian white noise processes with $\left\langle \xi_{x}\left(t\right)\xi_{y}\left(t^{\prime }\right)\right\rangle =\delta \left(t-t^{\prime }\right)\delta_{\mathrm{xy}}$.

We perform simulations integrating numerically the equations of motion in order to obtain the trajectory ${\vec{x}}_{i}$:

$$\begin{array}{c} \vec{a_{i}}={\vec{F}}_{i}, \end{array}$$

where ${\vec{â}}_{i}=\frac{d^{2}{\vec{x}}_{i}}{dt^{2}}$ is the acceleration, and we work with units of mass m=1. The values of the parameters in the model, shown in Table 1, have been selected in order to provide similar results to our experimental data, as described in *SI Appendix*.

**Table 1.** Parameters used in the standard model

Parameter	Description	Value
$k_{\text{rep}}$	Repulsive constant	$12.5\;\;{\text{s}}^{-2}$
$k_{\text{att}}$	Attractive constant	$5\;\;{\text{s}}^{-2}$
$d_{0}$	Equilibrium distance	5.8cm
μ	Alignment constant	$1.5\;\;{\text{s}}^{-1}$
$v_{0}$	Preferred speed	11cm/s
τ	Speed relaxation time	1.6s
$\sigma_{v}$	Noise constant in ${\hat{v}}_{i}$	6.4 cm/s^3/2^
$\sigma_{\varphi }$	Noise constant in ${\hat{\varphi }}_{i}$	2.6 cm/s^3/2^

We work in units of mass m=1.

Here, we work with N=39 individuals, matching the maximum number of fish in our experimental data. We solve the stochastic differential equation with the Euler–Maruyama algorithm (62). We choose random initial positions and velocities for the individuals such that the extension of the group and speeds are similar to the experimental school, but each individual is oriented uniformly at random. The simulation space is unbounded and positions can reach any value. We set a time step Δ*t*= 0.02 s and save all frames, analogous to the experimental data. We discard as transient the first 1000 frames and run simulations for 150,000 frames. We show in Movie S2 samples of trajectories generated by the standard model.

### Experimental Data.

Experiments, performed at the Scientific and Technological Centers UB (CCiTUB), University of Barcelona (Spain), were made with black neon tetra Hyphessobrycon herbertaxelrodi, a small freshwater fish of average body length 2.5 cm that has a strong tendency to form cohesive, highly polarized, and planar schools (63). Applicable institutional guidelines for the care and use of animals were followed. All procedures performed were in accordance with the standards approved by the Ethics Committee of the University of Barcelona.

Experiments consisted in N individuals freely swimming in a square tank of 100 cm of side with a water column of 5 cm of depth, resulting in approximately two-dimensional movement. Videos of the fish movement were recorded at 50 frames per second with a resolution of 5,312 to 2,988 pixels, such that the side of the tank measures 2,745 pixels. Digitized individual trajectories were obtained from the video recordings using the software idtracker.ai (64). Invalid values returned by the program caused by occlusions were corrected in a supervised way, semiautomatically interpolating with spline functions (now incorporated in the Validator tool from version 5 of idtracker.ai). For better accuracy, we projected the trajectories in the plane of the fish movement, warping the tank walls of the image into a proper square (*SI Appendix*). Moreover, we smooth the trajectories with a Gaussian filter with σ=2, truncating the filter at 5σ. We calculate the velocities and accelerations from the Gaussian filter using derivatives of the Gaussian kernel.

We performed experiments with N=39 fish (2 recordings of 60 min) and N=8 (6 recordings of 30 min). In Movie S1 we show a rendering of the evolution of the N=39 school with the digitized trajectories overlapped with the real video.

## Supplementary Material

Appendix 01 (PDF)

Movie S1. Rendering of the movement of fish in a experimental school of size N = 39, overlapped with the digitized trajectories, displayed in blue.

Movie S2. Dynamics for the standard model of schooling fish. The reference frame is comoving with the center of mass of the group. The position of the individuals and their velocities are shown in blue. We also display the different force terms in the model (legend): the alignment force (magenta), the attraction-repulsion force (red) and the friction-propulsion force (cyan). Each individual is linked with an edge to their Voronoi neighbours: in blue when they have attraction and in orange when they have repulsion.

Movie S3. Dynamics for the explicit anti-alignment model. The reference frame is comoving with the center of mass of the group. The position of the individuals and their velocities are shown in blue. We also display the different force terms in the model (legend): the alignment force (magenta), the attraction-repulsion force (red) and the friction-propulsion force (cyan). Each individual is linked with an edge to their Voronoi neighbours: in blue when they have attraction and in orange when they have repulsion.

Movie S4. Dynamics for the selective interactions model. The reference frame is comoving with the center of mass of the group. The position of the individuals and their velocities are shown in blue when they interact with other individuals and in black when they do not interact. We also display the different force terms in the model (legend): the alignment force (magenta), the attraction-repulsion force (red) and the friction-propulsion force (cyan). Each individual is linked to their Voronoi neighbours with an edge reaching half their distance if they are interacting: in blue when they have attraction and in orange when they have repulsion.

Movie S5. Sample of a recording where the focal fish i (green) tends to align with faster neighbours (blue) and ignore slower neighbours (red). Arrows indicate the velocity of individuals. Voronoi neighbours are coloured by their relative speed with the focal individual, *v_j_V* − v_i_.

## Data Availability

The experimental datasets can be accessed on Zenodo: https://zenodo.org/records/10890112 (65). The code to run the models and reproduce the figures is available on GitHub: https://github.com/Puco4/SelectiveSocialInteractions.
