# Supplementary material for: Selective social interactions and speed-induced leadership in schooling fish
Source: Proc Natl Acad Sci U S A. 2024 Apr 25;121(18):e2309733121. doi: 10.1073/pnas.2309733121 (PMC11067465; doi:10.1073/pnas.2309733121)
Supplement: Supplementary file 1 — Appendix 01 (PDF) [file pnas.2309733121.sapp.pdf]

SUPPLEMENTARY INFORMATION:

Selective social interactions and speed-induced leadership in schooling fish

Andreu Puy,[1,*] Elisabet Gimeno,[1,2] Jordi Torrents,[1,2] Palina Bartashevich,[3,4] M. Carmen Miguel,[2,5] Romualdo Pastor-Satorras,[1] and Pawel Romanczuk[3,4,6]

[1]*Departament de Física, Universitat Politècnica de Catalunya, Campus Nord B4, 08034 Barcelona, Spain*
[2]*Departament de Física de la Matèria Condensada, Universitat de Barcelona, Martí i Franquès 1, 08028 Barcelona, Spain*
[3]*Institute for Theoretical Biology, Humboldt-Universität zu Berlin, 10115 Berlin, Germany*
[4]*Excellence Cluster Science of Intelligence, Technische Universität Berlin, 10587 Berlin, Germany*
[5]*Institute of Complex Systems (UBICS), Universitat de Barcelona, Barcelona, Spain*
[6]*Bernstein Center for Computational Neuroscience, Berlin, 10099 Berlin, Germany*

STANDARD MODEL PARAMETERS SELECTION

Our aim with the standard model is to describe with few generic (linear) interaction terms the qualitative behaviour of schooling fish, rather than matching quantitative aspects. In this line of thought, instead of fitting the parameters to some goal function we use values at the expected order of magnitude comparing with the experimental data of schooling fish. We also confirmed that our qualitative results remain unchanged upon variation of these parameters.

The constants $k_{\rm rep}$ and $k_{\rm att}$ are chosen by comparing the pairwise attraction-repulsion force (Eq. 1) to the attraction-repulsion force map (Fig. 2a). In particular, we find $k_{\rm rep} > k_{\rm att}$, as fish may prioritize repulsion to attraction [1].

The equilibrium distance d_0 is fixed such that numerical simulations match the average distance between nearest neighbors observed in experimental data (Fig. 4b).

The alignment constant μ is taken analogously comparing the pairwise alignment force map (Eq. 2) to the alignment region in the alignment force map (Fig. 2b).

The fish preferred speed v_0 is chosen from the average individual speeds (Supplementary Fig. S10a).

The speed relaxation time τ is obtained from the average time period of the the burst-and-coast oscillations observed in the individual speeds.

The noise constants σ_v and σ_ϕ are chosen such that the explored regions in the force maps are comparable to the experimental data. Studying the PDFs of acceleration differences $\delta\vec{a}_i(t) \equiv \vec{a}_i(t+1) - \vec{a}_i(t)$, we found $\sigma_v > \sigma_\phi$, which we also preserve in the simulations.

TRAJECTORIES PROJECTION

The perspective of the camera was not exactly perpendicular to the plane of motion of the fish. For better accuracy, we project the calculated trajectories to the plane parallel to the bottom of the tank. In order to do this, first we look for the pixels of the corners of the tank walls in the videos and calculate the height and base of the corresponding rectangle in pixels. Because the tank is actually a square, we define the side of the tank L in pixels as the average value of the base and height of the rectangle image. We define the new tank corners: $(0,0)$, $(0,L)$, $(L,0)$ and (L,L) and we calculate the perspective transform matrix M from the original and new tank corners using the function `getPerspectiveTransform()` from the OpenCV library [2]. Finally, we obtain the transformed trajectories (x',y') applying a perspective transformation to the original trajectories (x,y), as explained in the OpenCV documentation:

$$(x',y') = \left(\frac{M_{11}x + M_{12}y + M_{13}}{M_{31}x + M_{32}y + M_{33}}, \frac{M_{21}x + M_{22}y + M_{23}}{M_{31}x + M_{32}y + M_{33}} \right).$$

[1] I. D. Couzin, J. Krause, R. James, G. D. Ruxton, and N. R. Franks, Collective Memory and Spatial Sorting in Animal Groups, Journal of Theoretical Biology **218**, 1 (2002).

[2] G. Bradski, The OpenCV Library, Dr. Dobb's Journal: Software Tools for the Professional Programmer **25** (2000).

* andreu.puy@upc.edu

Video S1. Rendering of the movement of fish in a experimental school of size $N = 39$, overlapped with the digitized trajectories, displayed in blue.

Video S2. Dynamics for the standard model of schooling fish. The reference frame is comoving with the center of mass of the group. The position of the individuals and their velocities are shown in blue. We also display the different force terms in the model (legend): the alignment force (magenta), the attraction-repulsion force (red) and the friction-propulsion force (cyan). Each individual is linked with an edge to their Voronoi neighbours: in blue when they have attraction and in orange when they have repulsion.

Video S3. Dynamics for the explicit anti-alignment model. The reference frame is comoving with the center of mass of the group. The position of the individuals and their velocities are shown in blue. We also display the different force terms in the model (legend): the alignment force (magenta), the attraction-repulsion force (red) and the friction-propulsion force (cyan). Each individual is linked with an edge to their Voronoi neighbours: in blue when they have attraction and in orange when they have repulsion.

Video S4. Dynamics for the selective interactions model. The reference frame is comoving with the center of mass of the group. The position of the individuals and their velocities are shown in blue when they interact with other individuals and in black when they do not interact. We also display the different force terms in the model (legend): the alignment force (magenta), the attraction-repulsion force (red) and the friction-propulsion force (cyan). Each individual is linked to their Voronoi neighbours with an edge reaching half their distance if they are interacting: in blue when they have attraction and in orange when they have repulsion.

Video S5. Sample of a recording where the focal fish i (green) tends to align with faster neighbours (blue) and ignore slower neighbours (red). Arrows indicate the velocity of individuals. Voronoi neighbours are coloured by their relative speed with the focal individual, $v_{jV} - v_i$.

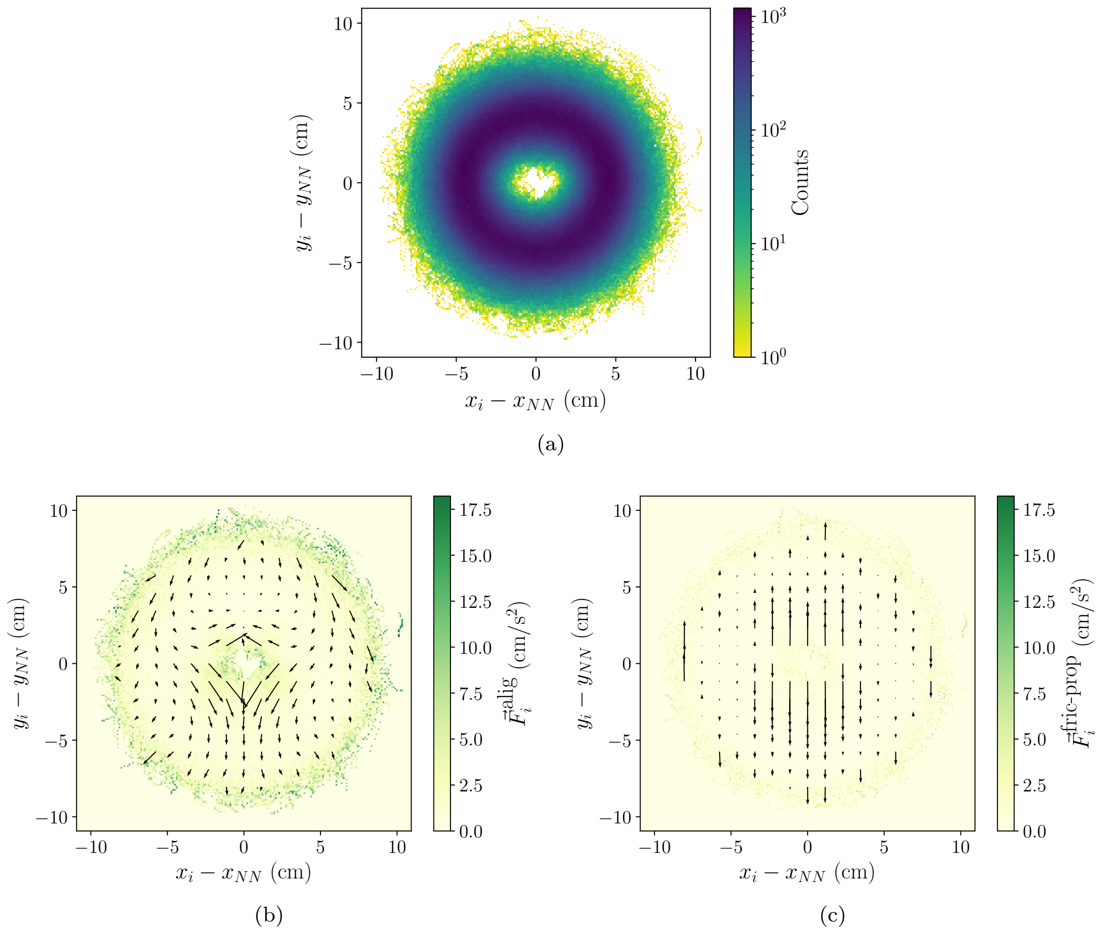

FIG. S1. (a) Counts, (b) average alignment and (c) average friction-propulsion force acting on an individual i depending on its relative position with nearest neighbour NN for the standard model. The y-coordinate is along the direction of motion of the individual. Forces are expressed in units of mass $m = 1$.

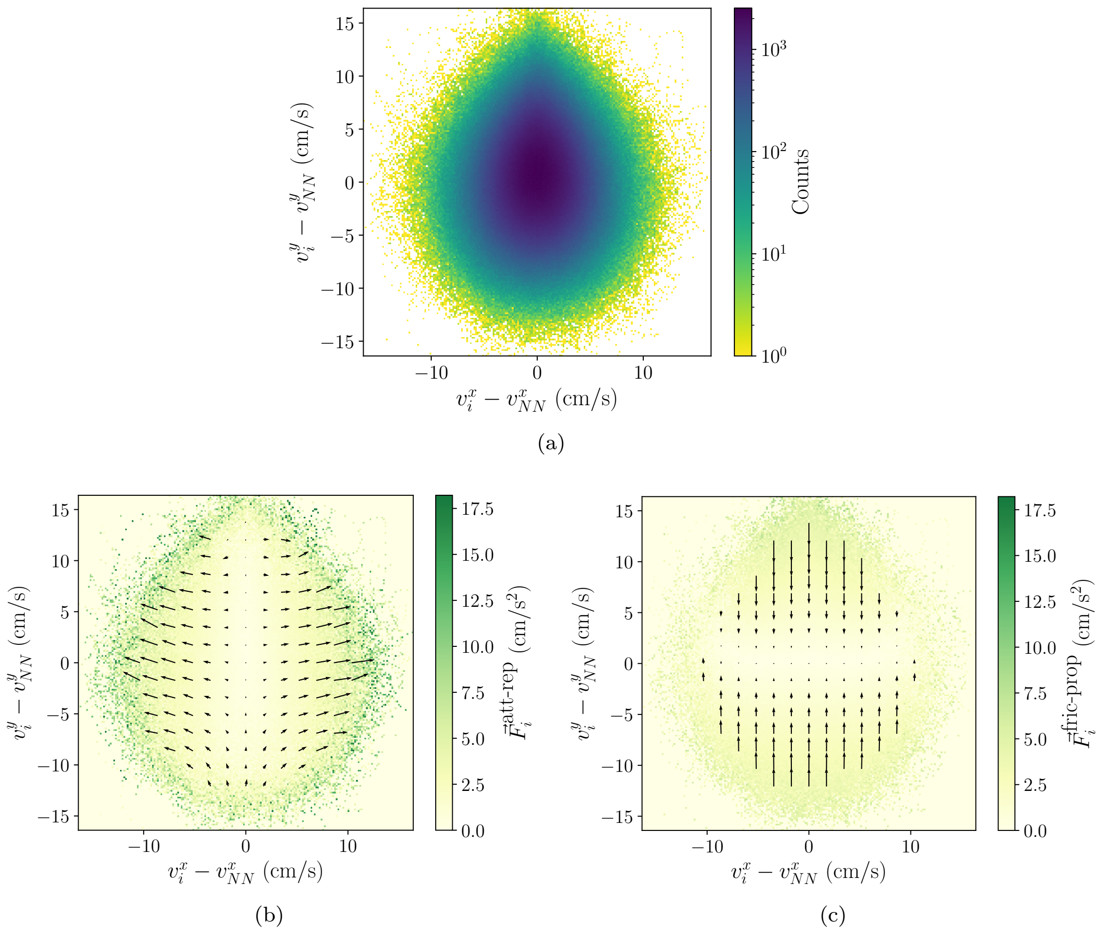

FIG. S2. (a) Counts, (b) average attraction-repulsion and (c) average friction-propulsion force acting on an individual i depending on its relative velocity with the nearest neighbour NN for the standard model. The y-coordinate is along the direction of motion of the individual. Forces are expressed in units of mass $m = 1$.

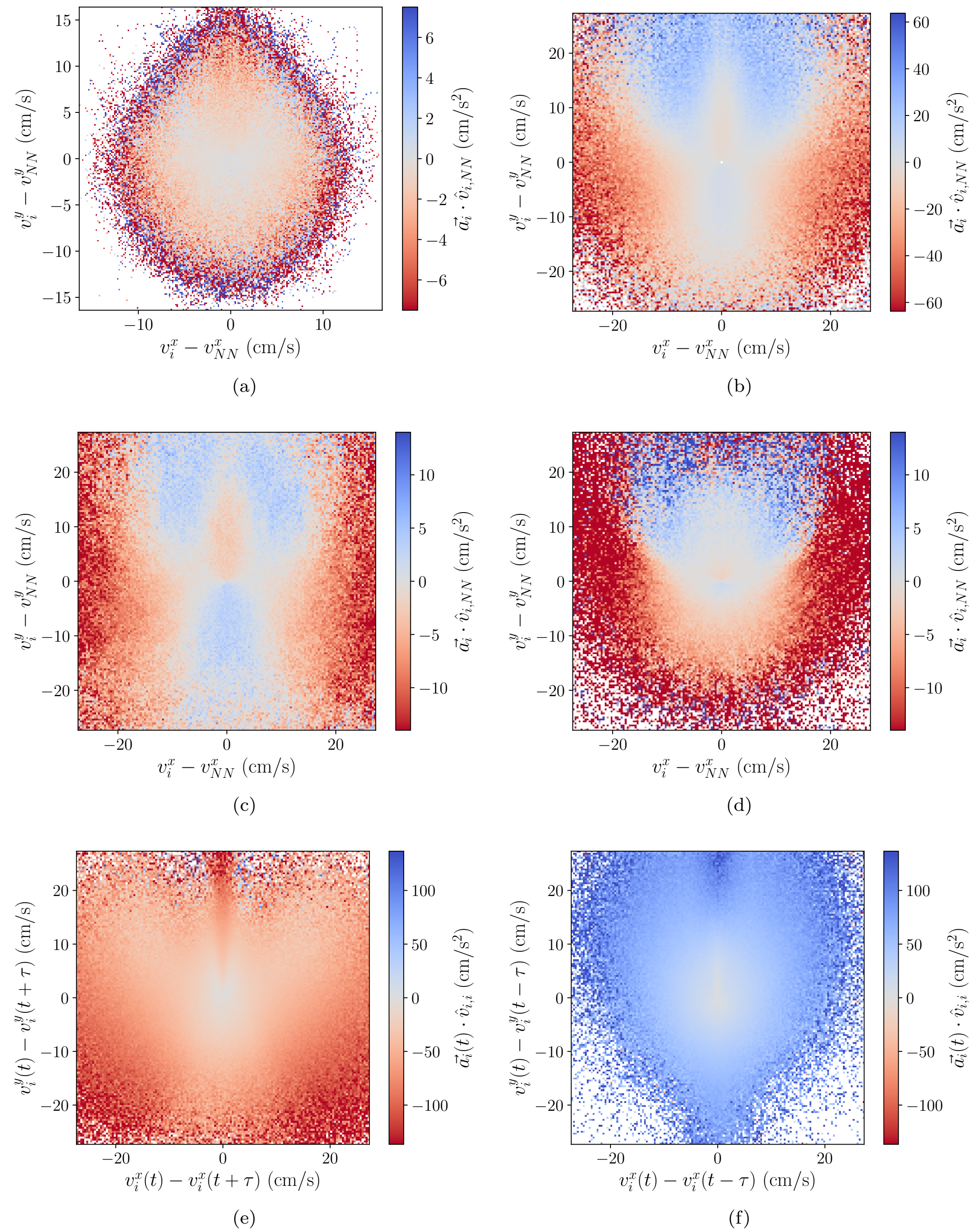

FIG. S3. Projection of the average acceleration along the radial direction of the alignment force map for (a) the standard model (Fig 1d), (b) the experimental data (Fig. 2b), (c) the explicit anti-alignment model (Fig. 3a), (d) the selective interactions model (Fig. 3b) and for the experimental data comparing the individual i with itself at a (e) positive (Fig. 5b) and (f) negative (Fig. 5c) delay of $\tau = 0.2$ s. In (a)-(d) the radial direction is given by the relative velocity of the individual with the nearest neighbour $\hat{v}_{i,NN} \equiv \frac{\vec{v}_i - \vec{v}_{NN}}{|\vec{v}_i - \vec{v}_{NN}|}$, while in (e)-(f) instead of the nearest neighbour we use the velocity of the individual i at the delayed time. In these plots, blue colors correspond to outward arrows (anti-alignment) in the alignment force map, and red colors to inward arrows (alignment).

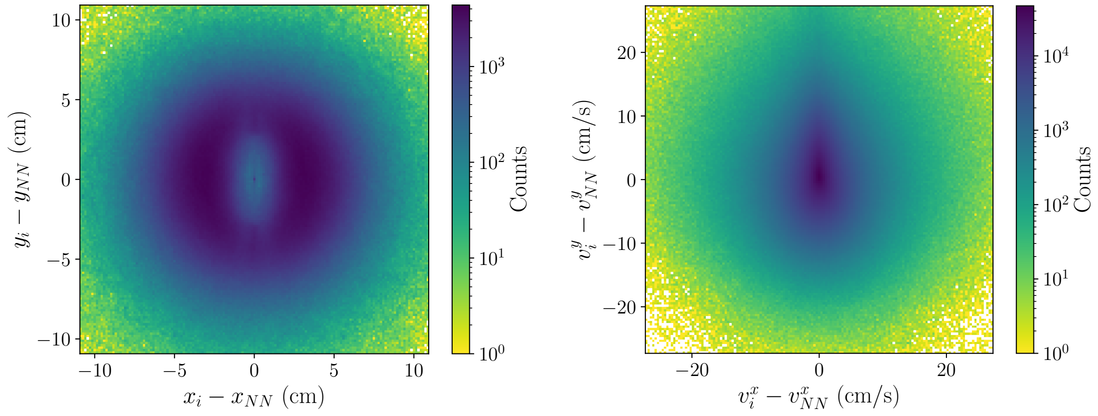

(a) (b)

FIG. S4. Counts for (a) attraction-repulsion and (b) alignment force maps for the experimental data.

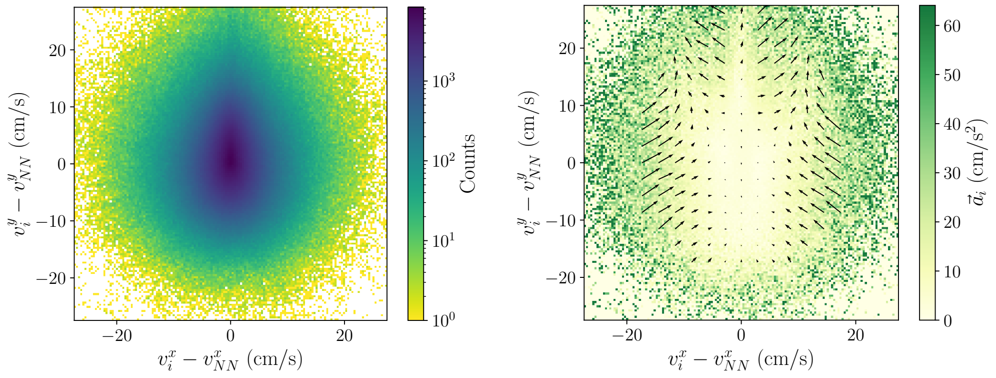

(a) (b)

FIG. S5. Alignment force maps for experimental data for a smaller number of fish $N = 8$.

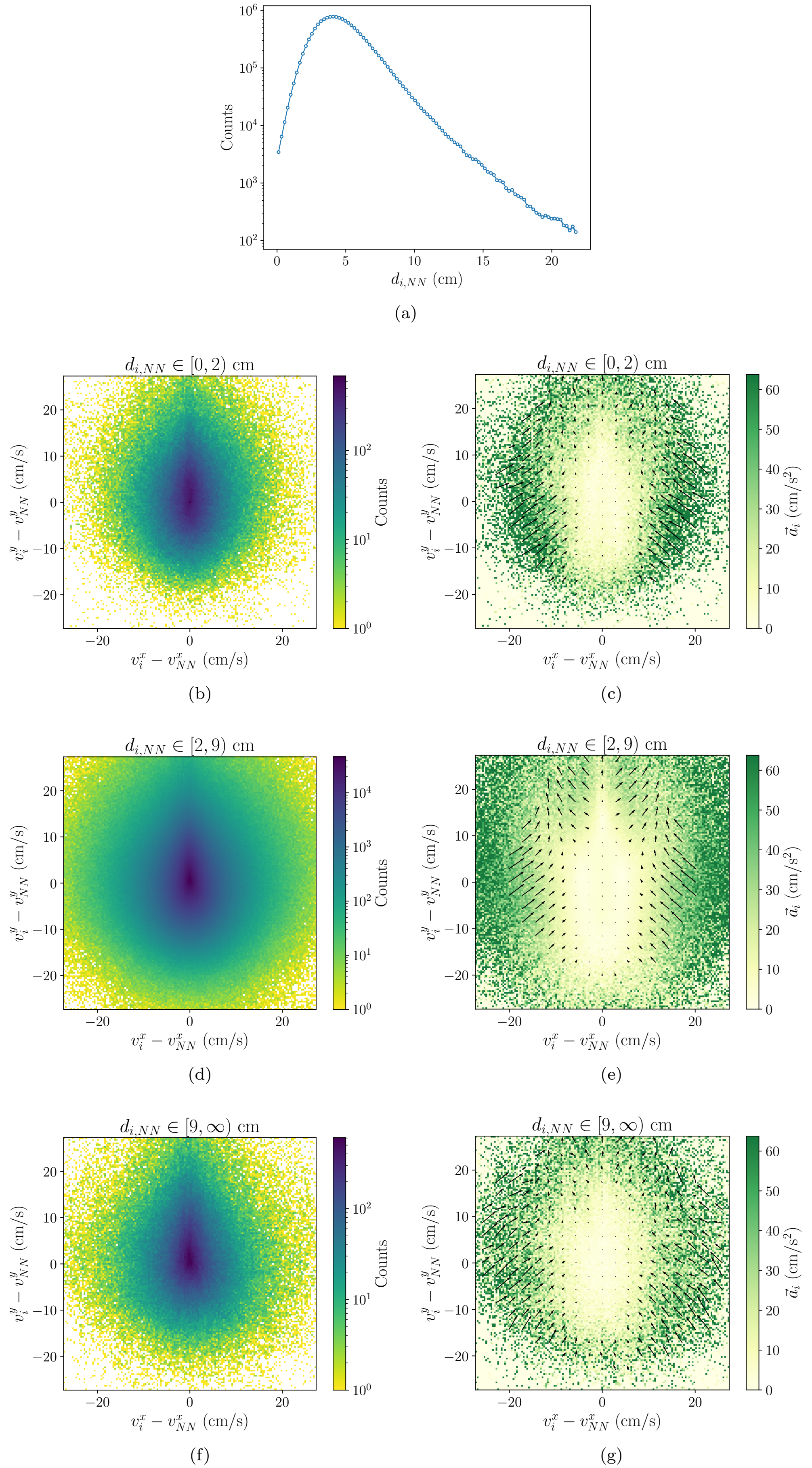

FIG. S6. (a) Distribution of distances between nearest neighbours $d_{i,NN}$ and (b)-(g) alignment force maps for different $d_{i,NN}$ for the experimental data.

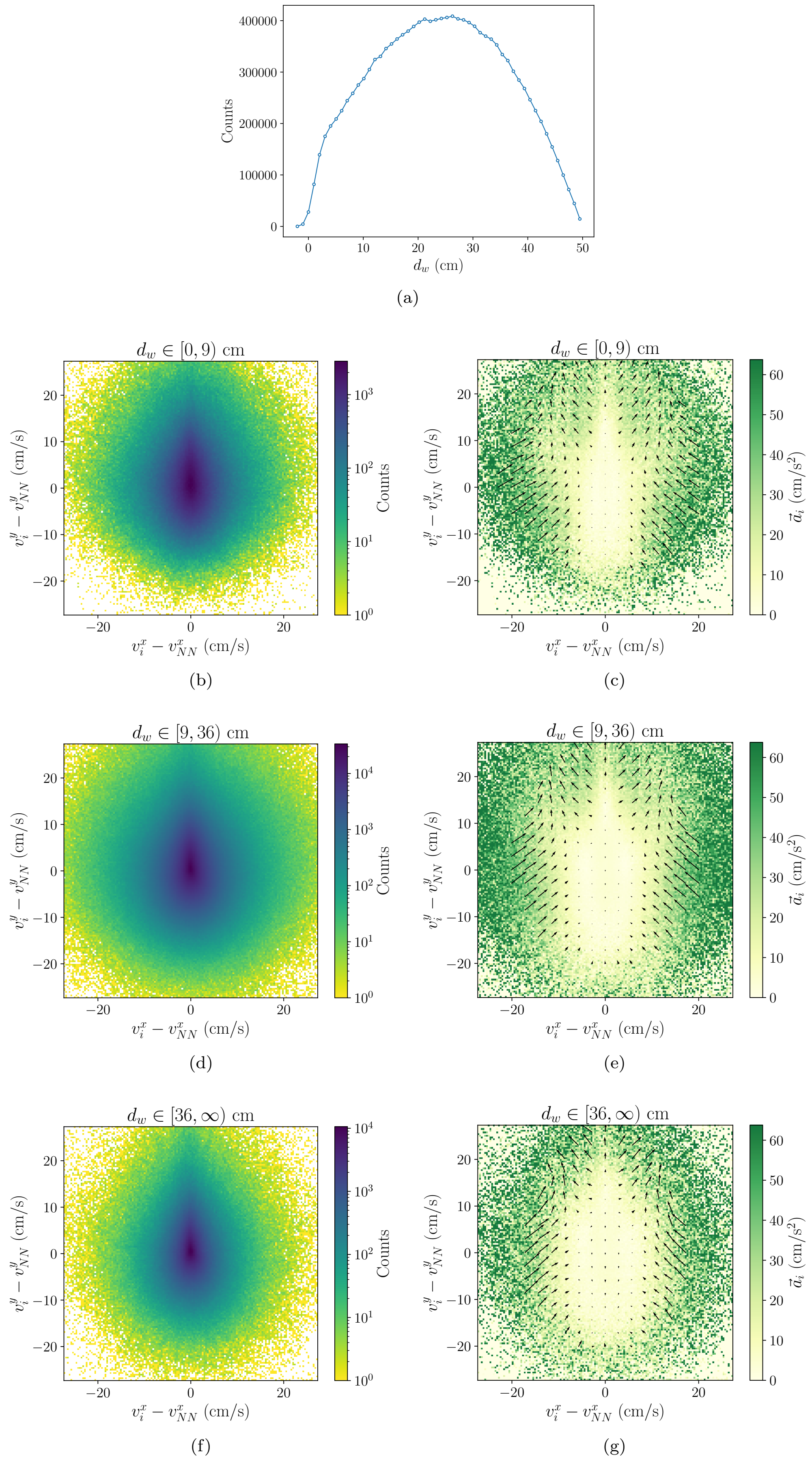

FIG. S7. (a) Distribution of minimum distances to the walls $d_w \equiv \min(x, L-x, y, L-y)$, for L the side of the tank, and (b)-(g) alignment force maps for different d_w of the individual i for the experimental data.

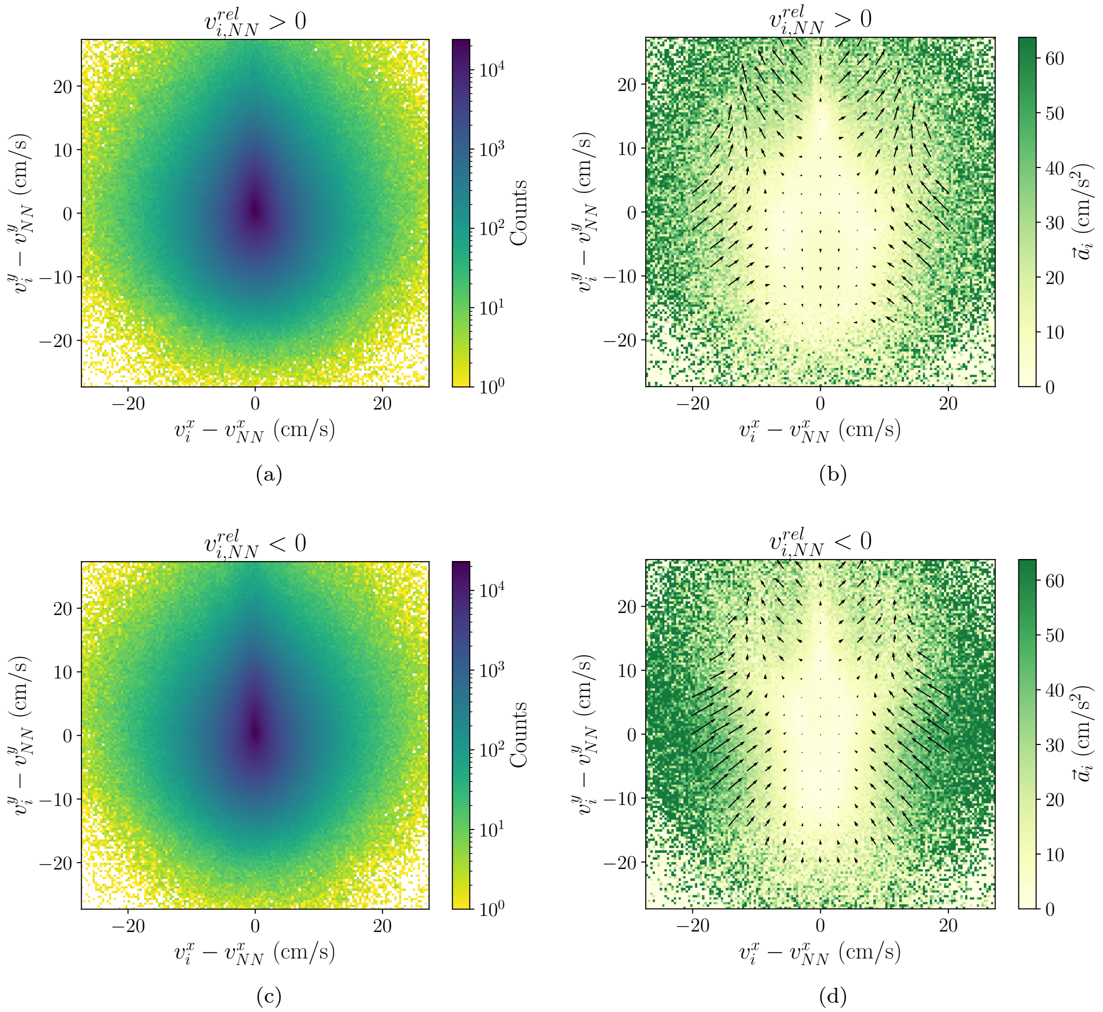

FIG. S8. Alignment force maps in the case the individual is approaching or moving away from the neighbour for the experimental data. We employ the relative velocity of the individual i with the nearest neighbour NN projected along the direction of their relative position, $v^{rel}_{i,NN} \equiv (\vec{v}_i - \vec{v}_{NN}) \cdot \frac{\vec{x}_i - \vec{x}_{NN}}{|\vec{x}_i - \vec{x}_{NN}|}$. In (a), (b) $v^{rel}_{i,NN} > 0$ and the individual is moving away from the neighbour. In (c), (d) $v^{rel}_{i,NN} < 0$ and the individual is approaching the neighbour. We notice in this last case the alignment region is slightly stronger.

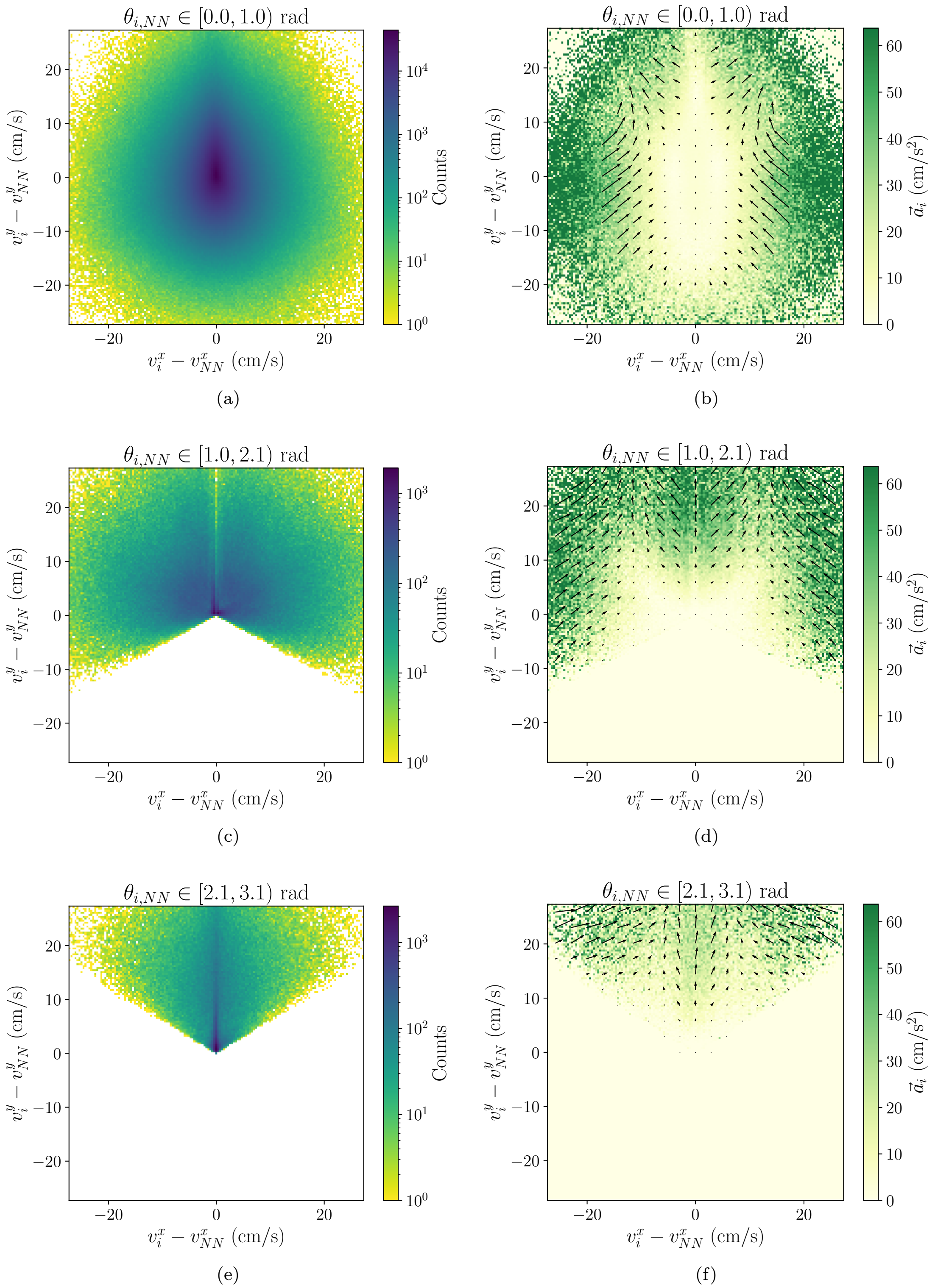

FIG. S9. Alignment force maps for different heading orientations $\theta_{i,NN}$ between an individual i and its nearest neighbour NN for the experimental data.

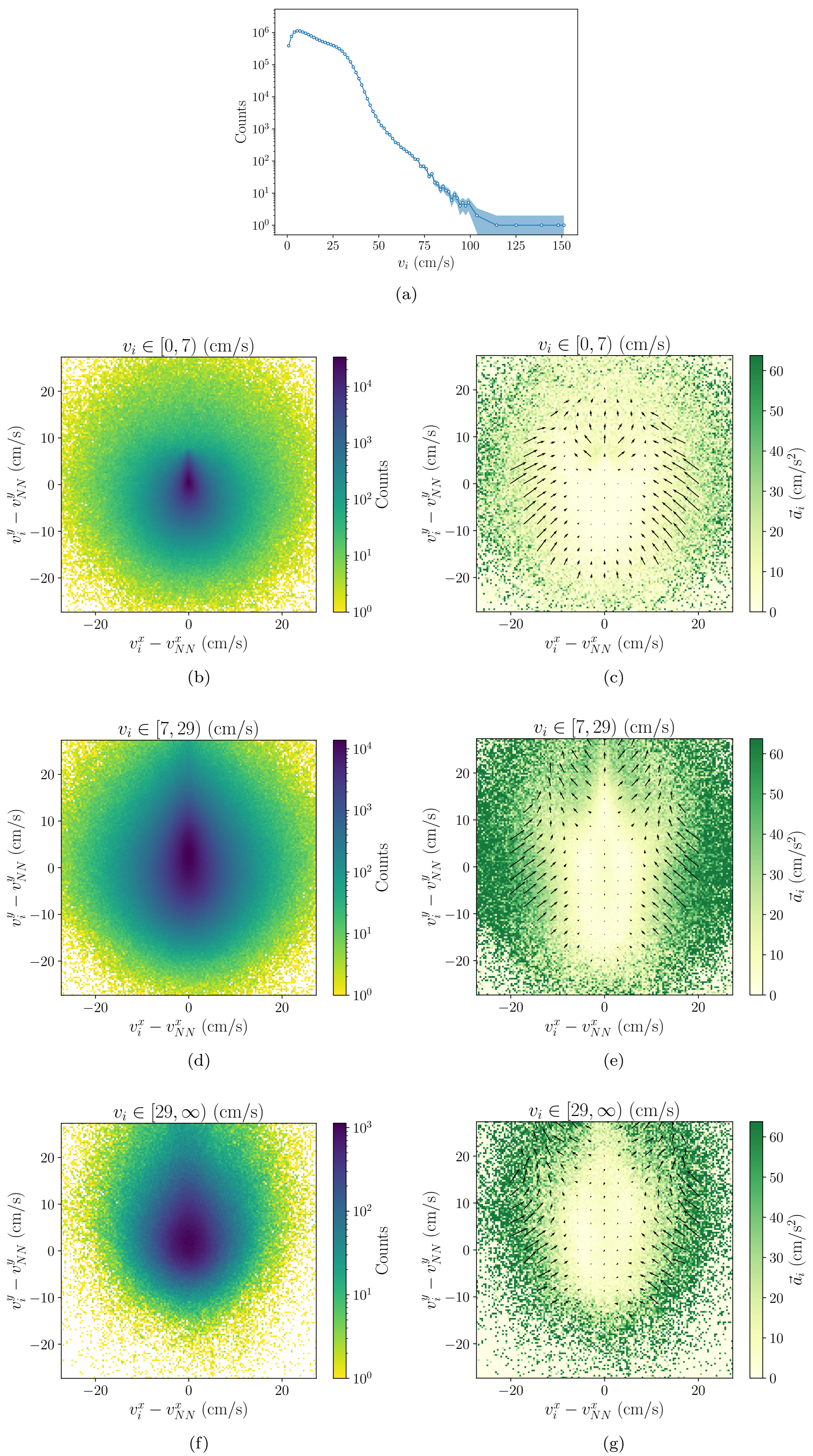

FIG. S10. (a) Distribution of individual speeds v_i and (b)-(g) alignment force maps for different v_i for the experimental data. Notice how the strength of the force increases with the speed of the individual and the anti-alignment region shifts to the front. The error band in (a) is calculated from the standard deviation of a Bernoulli distribution with the probability given by the fraction of counts in each bin.

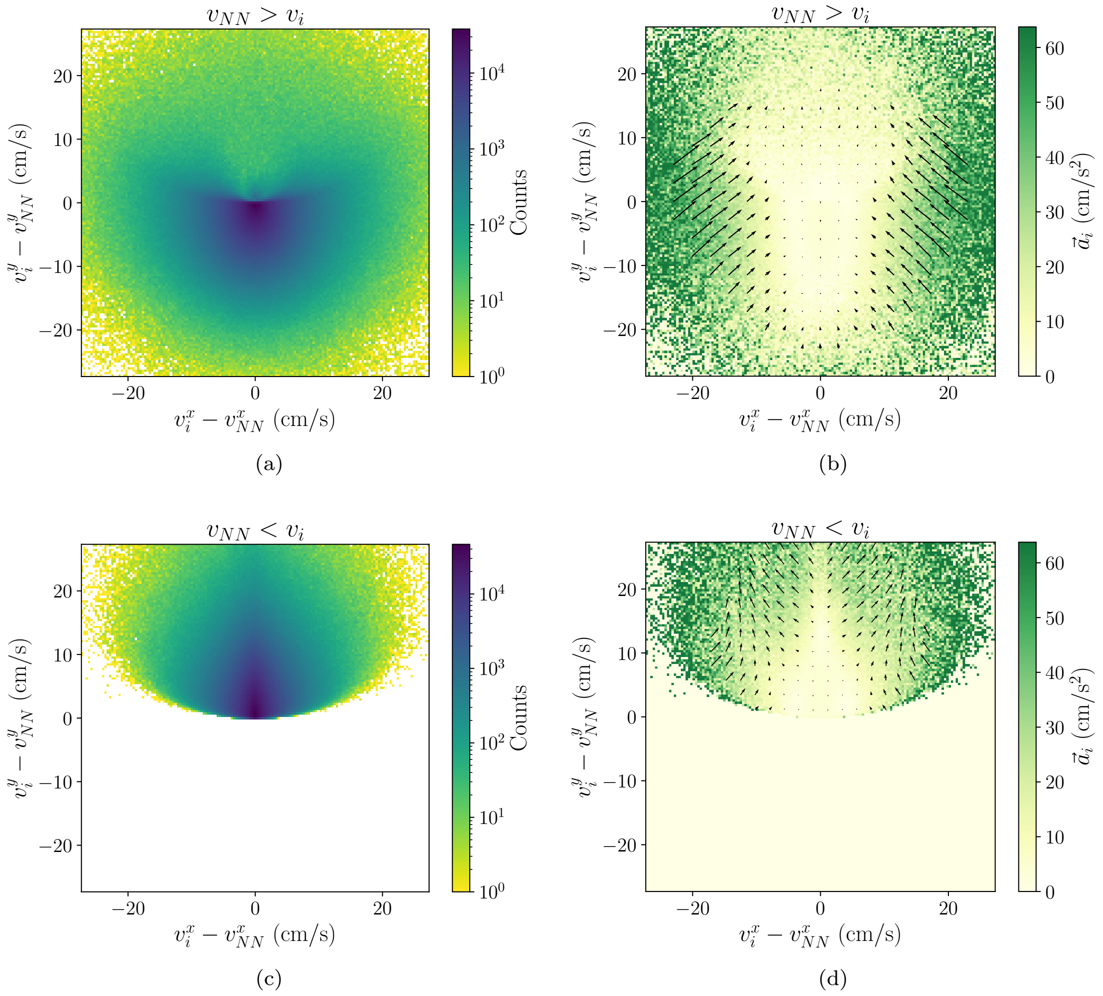

FIG. S11. Alignment force maps for different relative velocities between the neighbour and the individual for the experimental data. They essentially occupy excluded regions in the force map.

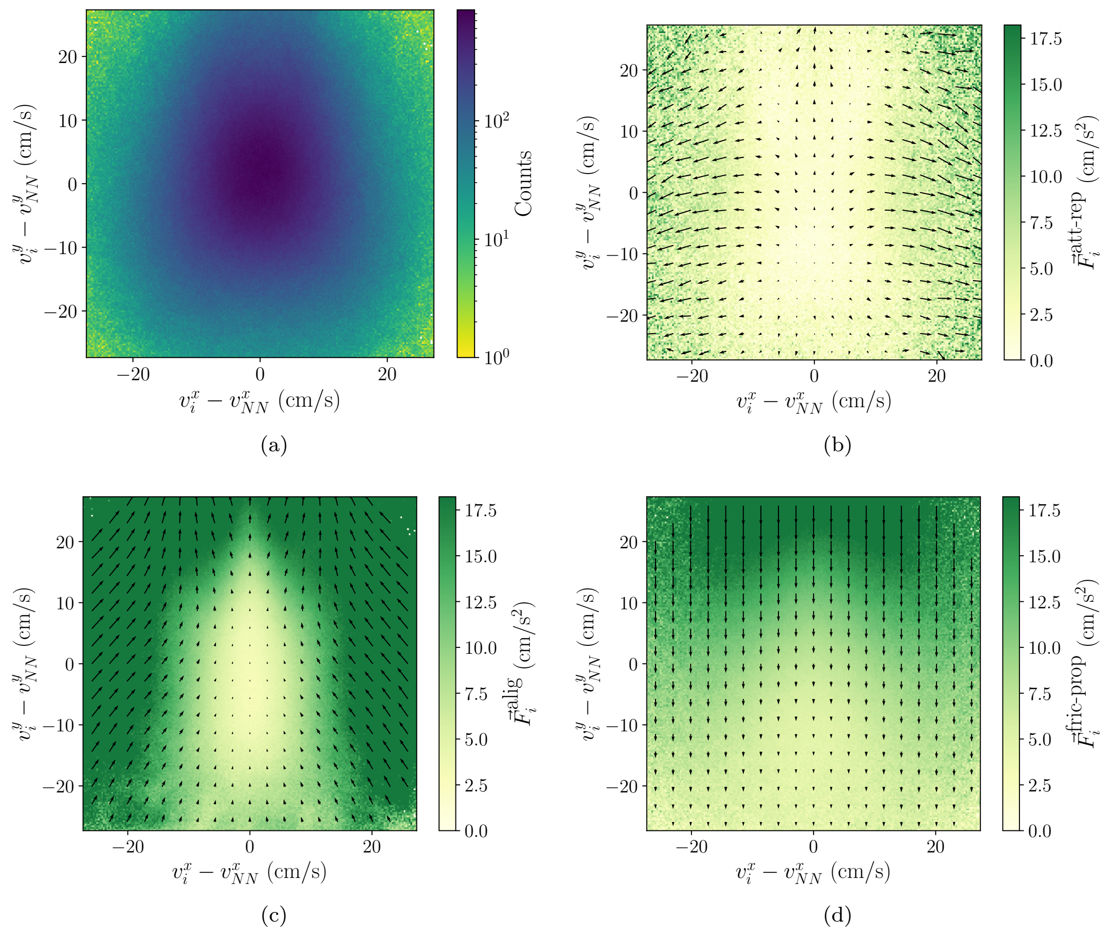

FIG. S12. (a) Counts, (b) average attraction-repulsion, (c) average alignment and (d) average friction-propulsion force acting on an individual i depending on its relative velocity with the nearest neighbour NN for the explicit anti-alignment model.

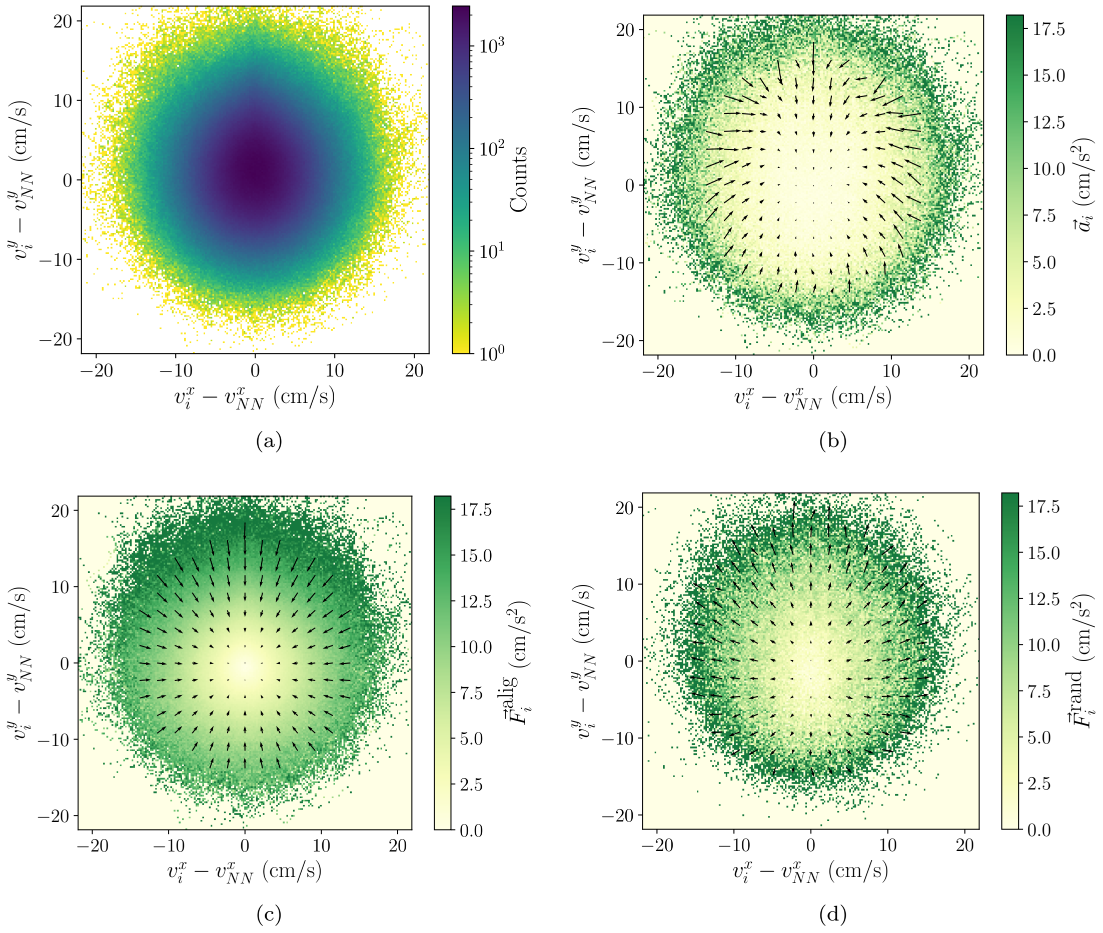

FIG. S13. (a) Counts, (b) average acceleration (alignment force map), (c) average alignment force and (d) average persistent random force acting on an individual i depending on its relative velocity with the nearest neighbour NN for the standard model with a persistent random force. Here we use an individual has a probability rate $r = 1/20$ to switch off the social forces and experience a persistent random force of amplitude $A = 1.5$ for a time $\tau = 5$. The persistent random force appears as an effective anti-alignment force with arrows pointing outwards. However, in this regime the alignment force dominates and the acceleration is displayed with alignment with arrows pointing inwards.

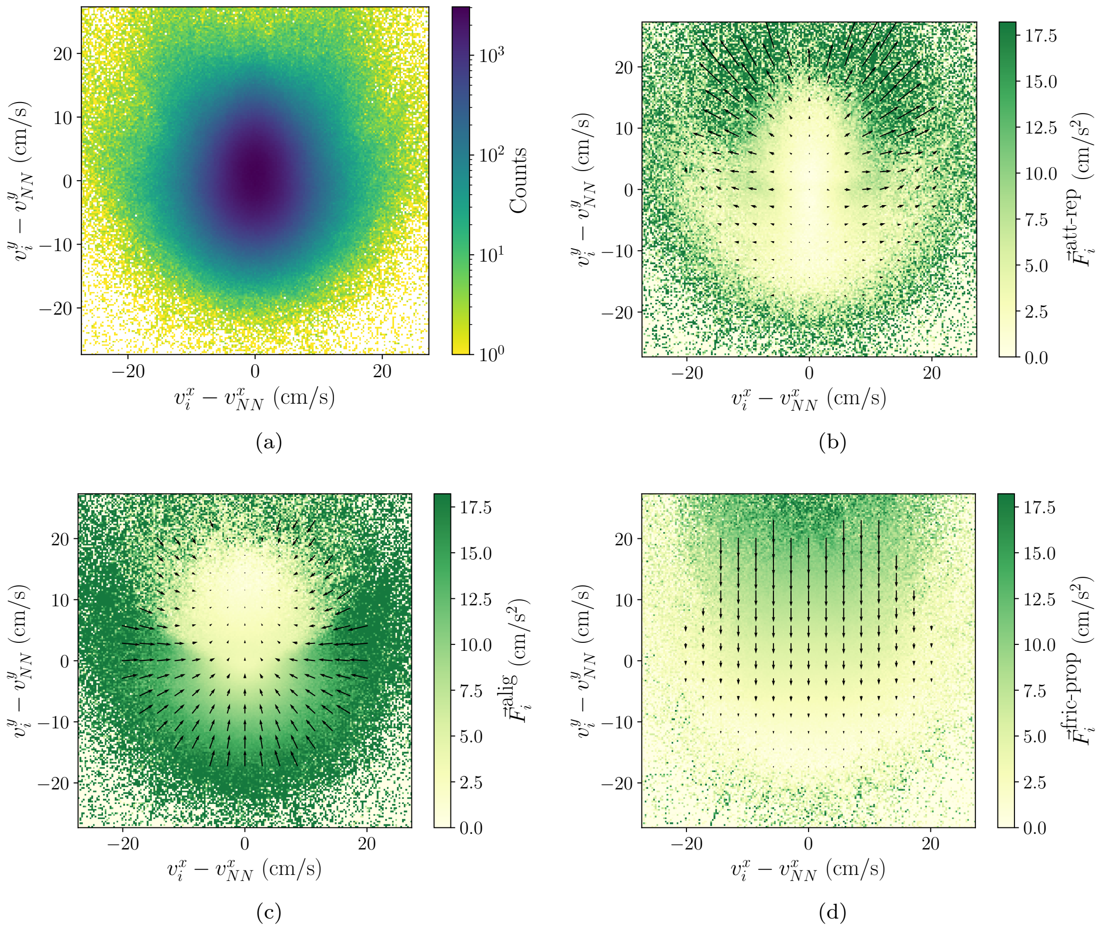

FIG. S14. (a) Counts, (b) average attraction-repulsion, (c) average alignment and (d) average friction-propulsion force acting on an individual i depending on its relative velocity with the nearest neighbour NN for the selective interactions model. Here the attraction-repulsion force for slower nearest neighbours acts as an effective anti-alignment force with arrows pointing outwards.

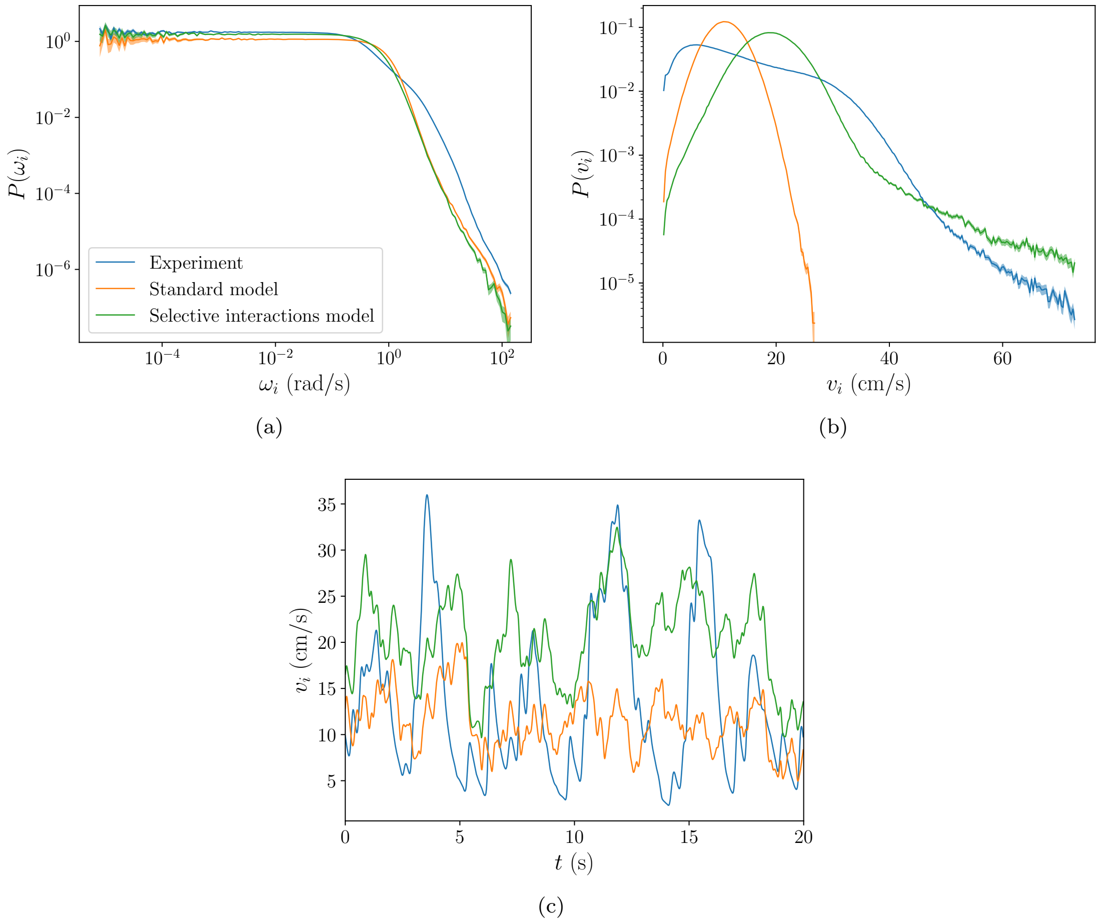

FIG. S15. (a) PDF of the turning rate ω_i, (b) PDF of the speed v_i and (c) sample of the temporal evolution of an individual speed for the experimental data, the standard model and the selective interactions model.

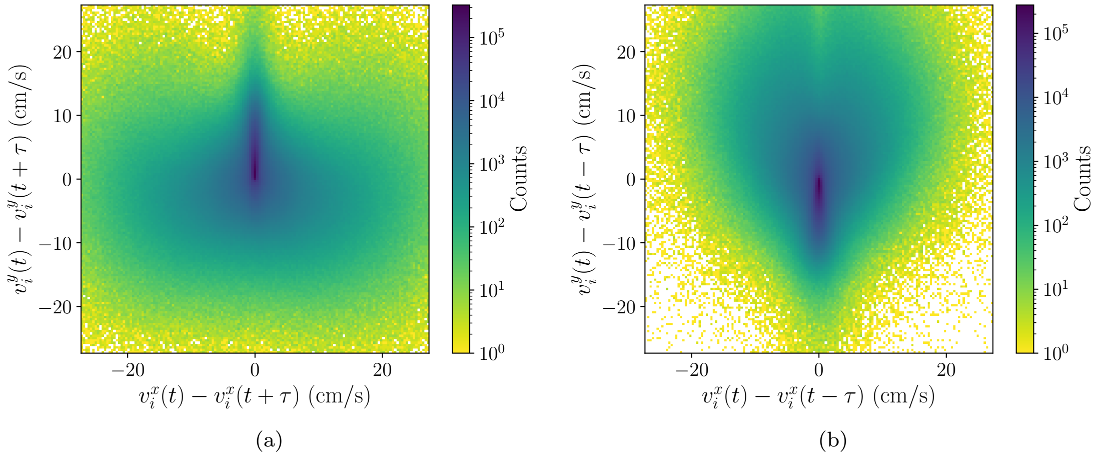

FIG. S16. Counts for the alignment force maps for the experimental data comparing the individual at present with itself at a (a) positive and (b) negative delay with $\tau = 0.2$ s.

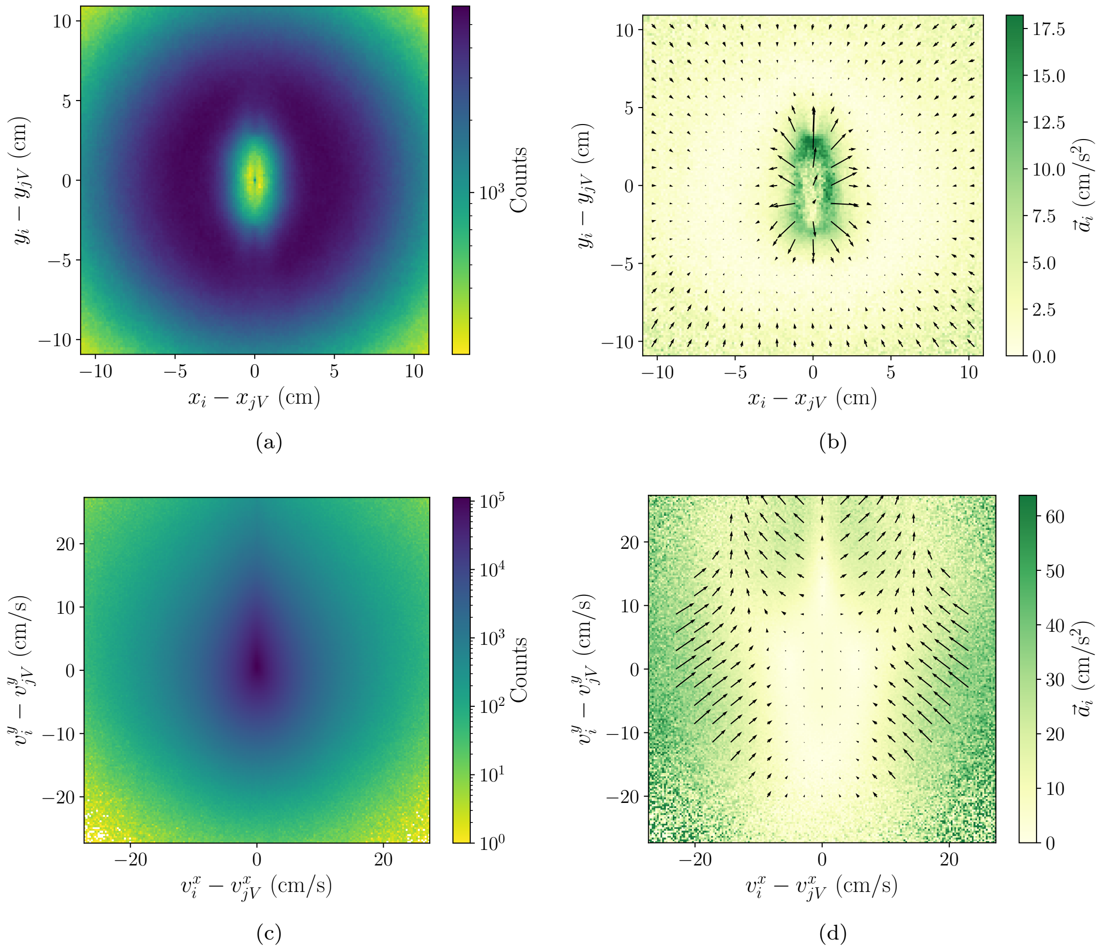

FIG. S17. For Voronoi neighbours: (a) counts of the relative positions between neighbours, (b) attraction-repulsion force map, (c) counts of the relative velocities between neighbours and (d) alignment force map for the experimental data.

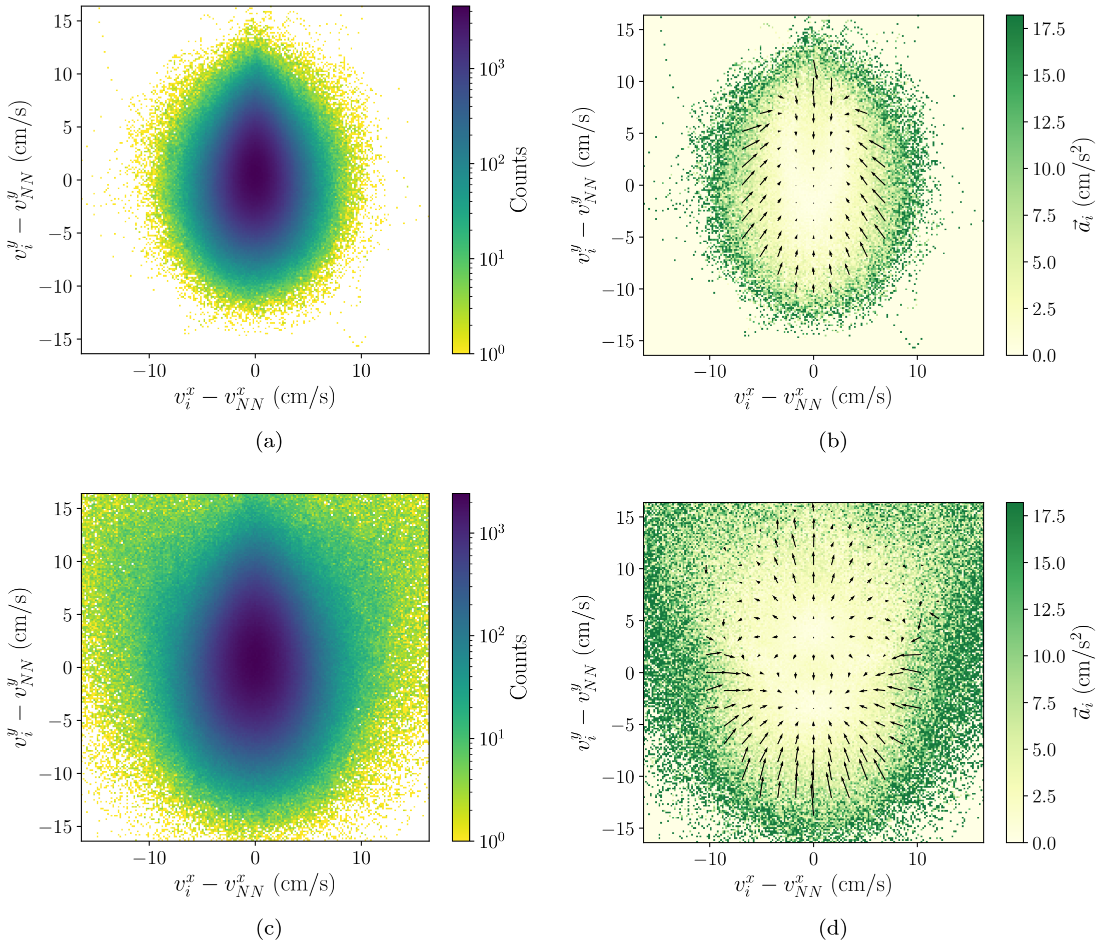

FIG. S18. Simulations where individuals interact socially only with the nearest neighbour: (a) counts and (b) alignment force map for the standard model, (c) counts and (d) alignment force map for the selective interactions model.
